# Inclusion Scenarios and Conformational Flexibility of the SSRI Paroxetine as Perceived from Polymorphism of β-Cyclodextrin–Paroxetine Complex

**DOI:** 10.3390/ph15010098

**Published:** 2022-01-14

**Authors:** Thammarat Aree

**Affiliations:** Department of Chemistry, Faculty of Science, Chulalongkorn University, Bangkok 10330, Thailand; athammar@chula.ac.th; Tel.: +66-2-2187584; Fax: +66-2-2187598

**Keywords:** β-cyclodextrin, paroxetine, selective serotonin reuptake inhibitors (SSRIs), inclusion complex, polymorphism, X-ray analysis, DFT calculation

## Abstract

Depression, a global mental health problem, is prevalent during the coronavirus disease 2019 (COVID-19) pandemic and can be efficiently treated by selective serotonin reuptake inhibitors (SSRIs). Our study series aims at forwarding insights on the β-cyclodextrin (β-CD)–SSRI inclusion complexes by X-ray crystallography combined with density functional theory (DFT) calculation. Here, we report a new crystal form (II) of the 1:1 β-CD–paroxetine (PXT) complex, which is inspired by the reported 2:1 β-CD–PXT complex (crystal form I), reflecting an elusive phenomenon of the polymorphism in CD inclusion complexes. The β-CD–PXT polymorphism stems from the PXT conformational flexibility, which is defined by torsion angles *κ*, *ε* around the -CH_2_–O- group bridging the A- and C–D-rings, of which those of PXT in I and II are totally different. While PXT (II) in an open V-shaped conformation that has the B-ring shallowly inserted in the β-CD cavity, PXT (I) in a closed U-shaped structure is mostly entirely embedded in the β-CD dimeric cavity, of which the A-ring is deeply inserted in the main β-CD cavity. However, PXT molecules in both crystal forms are similarly maintained in the CD cavity via host–guest N–H···O5/O6 H-bonds and C/O–H···π(B/C) interactions and β-CDs have similar 3D arrangements, channel (II) vs. screw-channel (I). Further theoretical explorations on the β-CD–PXT thermodynamic stabilities and the PXT conformational stabilities based on their potential energy surfaces (PESs) have been completed by DFT calculations. The 2:1 β-CD–PXT complex with the greater presence of dispersion interactions is more energetically favorable than the unimolar complex. Conversely, whereas free PXT, PXT (II) and PXT in complex with serotonin transporter are more energetically stable, PXT (I) is least stable and stabilized in the β-CD cavity. As SSRIs could lessen the COVID-19 severity, the CD inclusion complexation not only helps to improve the drug bioavailability, but also promotes the use of antidepressants and COVID-19 medicines concurrently.

## 1. Introduction

Depression is a common mental illness worldwide, affecting 3.8% of the population or ~280 million people, and 5.0% of adults suffer from it [1]. The emerging coronavirus disease 2019 (COVID-19) made 1 in 3 adults depressed from the pandemic [2]. The COVID-19 pandemic caused global havoc not only on human lives—due to the rapid infections and high mortality rate of infected people (~3.4% in March 2020 [3] and ~2% in November 2021 after mass vaccination [4])—but also on the economics disruption due to the sudden, long shutdown. The pandemic had a domino effect on the mental health problem, in particular, stress and depression. Good news happened in the midst of the COVID-19 pandemic. Recent studies have added to the growing body of evidence that antidepressants could lessen the risk of death or intubation in patients hospitalized for COVID-19. This is due to small drug–drug interactions of COVID-19 medications and antidepressants, based on cytochrome P450 metabolism [5], and the antidepressant anti-inflammatory effects in lung disease, thus facilitating the combinatory treatment of both drugs [6,7,8,9,10].

**Scheme 1:**
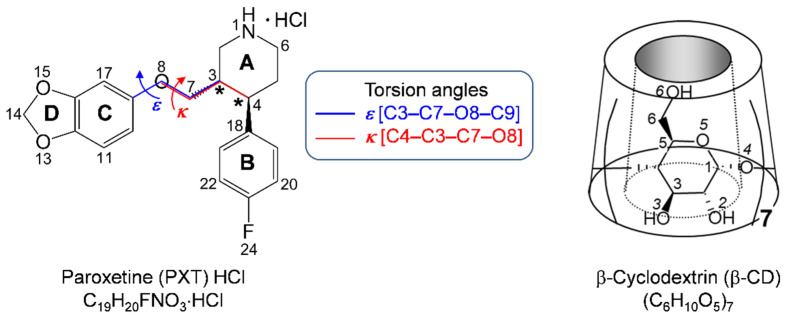
Chemical structures and atom numbering schemes of PXT and β-CD; the A-, B-, C-, D-rings and chiral centers of PXT are marked. The two key torsion angles *κ*, ε describing the PXT conformational flexibility are emphasized. Note that PXT, (3*S*,4*R*)-(–)-3-[(1,3-benzodioxol-5-yloxy)methyl]-4-(4-fluorophenyl)piperidine, is marketed in the hydrochloride (HCl) salt form for enhanced solubility and bioavailability. For β-CD, C and O atoms are respectively indicated by normal and italic numbers.

Depression can be efficiently treated by selective serotonin reuptake inhibitors (SSRIs)—second generation—and tricyclic antidepressants (TCAs)—first generation medications. The antidepressants approved by the US Food and Drug Administration [11] include, for example, SSRIs—escitalopram (Lexapro), fluoxetine (Prozac), sertraline (Zoloft)—and TCAs—amitriptyline (Elavil), doxepin (Sinequan), imipramine (Tofranil). Paroxetine (Paxil; PXT), a family member of SSRIs, comprises the piperidine A-ring, which is linked at C3 via the C7–O8 bridge to the 1,3-benzodioxole moiety (C–D-rings) and is connected at C4 to the 4-fluorophenyl (B-ring). Hence, PXT without a side chain is the bulkiest and highly conformationally flexible SSRI; see the two key torsion angles *κ*, *ε* in Scheme 1. While SSRIs are structurally diverse, TCAs are structurally similar. Both drug types have equivalent efficacy in treating depression. TCAs have a drawback of higher side effects compared to SSRIs due to their less-specific binding to the serotonin transporter (SERT). However, the uses of both TCA and SSRI drugs are limited due to their low water solubility and undesired side effects. To improve the drug bioavailability, cyclodextrin encapsulation is a suitable method, as demonstrated for TCAs [12,13] and SSRIs [14,15,16,17].

Cyclodextrins (CDs) have been known to us longer than 130 years since their first publication by Villiers in 1891, as reviewed by [18]. They are obtained by enzymatic degradation of starch, thus comprising 6–8 glucose units, namely, α-, β- and γ-CDs, respectively. CDs resemble an empty, truncated cone with amphipathic properties—hydrophilic perimeters and apolar central nanocavity, which is suitable for anchoring different guest molecules, forming inclusion complexes [18], Scheme 1. CD inclusion complexation has a wide spectrum of applications. Even though there is a plethora of reviews and books on the applications of CD inclusion complexes, given here are the more recent references on pharmaceutics, cosmetics, medicine, food, chromatography, biotechnology and nanotechnology [18,19,20,21,22]. In pharmaceutical technology, CD encapsulation improves the physicochemical and pharmacological properties of drugs, in particular, antidepressants [23]. Although pharmaceuticals tend to exhibit polymorphism [24], this phenomenon remains elusive for CD inclusion complexes. This is due to the specific host–guest arrangements and interactions both in the asymmetric units and crystal lattices, resulting in distinct crystal packing patterns. The given examples are β-CD–benzoic acid [25] and β-CD–4-phenylpyridine-*N*-oxide [26]. Polymorphism plays a pivotal role in the improvement of physicochemical properties and bioavailability of solid drugs [27,28,29].

For the past five years, we have launched a series of comprehensive structural studies by single-crystal X-ray diffraction combined with density functional theory (DFT) calculation for the atomic-level understanding of the β-CD–antidepressant inclusion complexes. We have unambiguously unveiled the nature and characteristics of inclusion complexation of β-CD with TCAs [30,31,32,33] and SSRIs [34]. Although TCAs and SSRIs do not share structural similarities, and they complex with β-CD at different host–guest ratios, their inclusion structures are in common with the aromatic ring embedded in the CD cavity and are similarly stabilized by C–H···π interactions. The presence of guest–guest halogen···halogen interactions facilitate the 2:2 β-CD–sertraline/fluoxetine HCl inclusion complexation [34]. On the other hand, without intermolecular halogen···halogen interactions, the β-CD–TCA HCl complexes exclusively exist in a unimolar ratio [30,31,32,33]. As the structural components are extended from 2–3 rings in sertraline/fluoxetine to 4 rings in PXT, the bulkier drug molecule adapts its structure to a unique U-shaped conformation for total inclusion in the β-CD dimeric cavity, yielding a 2:1 host–guest complex [35]. The improved thermodynamic stabilities of the β-CD–antidepressant complexes perceived by DFT calculations help to lessen side effects and enhance the bioavailability of drugs [33,34].

Additionally, we have provided a thorough structural comparison of three key SSRI drugs (sertraline, fluoxetine and PXT) covering three distinct lattice environments, i.e., from (i) the uncomplexed form (freebase/HCl), (ii) the drug formulated in the CD cavity (carrier) for delivery and to (iii) the drug in action while optimally interacting with the surrounding amino acids around a protein binding pocket. The larger root mean square (rms) fits (i.e., the greater structural differences) are obtained when comparing the drugs bound to proteins with those in other circumstances. This suggests that the pharmacological functions of drugs require conformational adaptability for optimal binding to target proteins [36], as demonstrated for TCAs (nortriptyline, amitriptyline, clomipramine and doxepin, [30,32] and SSRIs (sertraline, fluoxetine and PXT) [34].

The X-ray structure of the 2:1 β-CD–PXT base complex [35] differs from the quite stable NMR structure of the 1:1 β-CD–PXT base with an association constant of ~2000 M^−1^ and the C–D-rings are deeply inserted in the CD cavity [37]. The distinction of solid and solution structures of the β-CD–PXT base, the host–guest size complementarity (the β-CD height and diameter of ~7.8 Å vs. the six-membered A/B/C ring size of ~4.6 Å) and the bulkiest and least rigid drug (among SSRIs) inspired two hypotheses for this work. (i) We envisage that the 1:1 or 2:2 host–guest ratio might plausibly exist, as both the B- and C–D-rings could competitively bind to the β-CD cavity. (ii) Because the flexible PXT is conformationally forced to some extent while confining in the β-CD cavity or binding to protein pockets for optimum complexation [35,38], other stable conformations are plausible. To validate the two hypotheses, we attempt to crystallize the concentrated β-CD–PXT HCl solution for single-crystal X-ray diffraction analysis, evaluate the complex thermodynamic stabilities and rationalize the PXT conformational flexibility via an exploration of the potential energy surface (PES) by DFT calculations.

## 2. Results and Discussion

**Figure 1:**
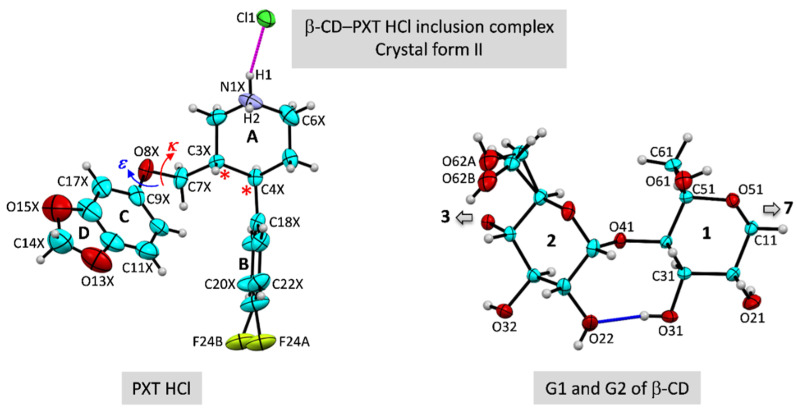
Atom numbering schemes of (3*S*,4*R*)-PXT HCl and β-CD in crystal form II of the β-CD–PXT HCl complex; ORTEP plot at 30% probability level. The PXT molecule is protonated at the N1X axial position (H2) and counterbalanced, equatorially coordinated (H1) by a fully occupied chloride. The chiral centers C3X and C4X in the A-ring are red starred. The connecting blue and magenta lines respectively indicate the intramolecular, interglucose O3(*n*)∙∙∙O2(*n* + 1) hydrogen bonds stabilizing the round β-CD conformation and the intermolecular N1X–H1∙∙∙Cl1 H-bond in PXT HCl.

We employ the conventional carbohydrate nomenclature for CDs, i.e., atoms C62–O62A(B) represent the methylene C6–H_2_ linked with the twofold disordered hydroxyl O6–H groups (sites A and B) of glucose residue 2 (G2) in the 1:1 β-CD–PXT HCl complex (form II), Figure 1. For the 2:1 β-CD–PXT base inclusion complex (form I [35]), two β-CDs are additionally numbered 1 and 2. The atom numbering of PXT is assigned according to its IUPAC name and is arbitrarily labeled with letters P and X for forms I and II, respectively. For a better understanding of this work, we organize the results and discussion as follows. Section 2.1 and Section 2.2, respectively, deal with the structural adaptation of host β-CD and guest PXT upon inclusion complexation, demonstrating the induced-fit process. Section 2.3 illustrates the rare phenomenon of polymorphism in the β-CD-PXT inclusion complex (forms I and II). The last two sections are allocated for the theoretical insights by DFT calculations: Section 2.4—the significant role of dispersion interactions in thermodynamic stabilities of the 1:1 and 2:1 complexes, and Section 2.5—first-time deep exploration of the PXT conformational flexibility in various lattice environments through a potential energy surface.

### 2.1. Marginal β-CD Structural Changes upon Inclusion of PXT

The conformationally flexible PXT is not only primarily responsible for the polymorphism of the β-CD–PXT complex (see Section 2.3, Section 2.4 and Section 2.5), but also induces the β-CD structural changes to the extent described below. To quantify the CD conformational changes upon the drug inclusion, we structurally compare a CD pair at both microscopic (elemental geometrical parameters) and macroscopic (a global parameter—rms fit) levels.

The CD structure comprises six parameters that can be categorized into three groups (Table S2). Group 1: the parameters relevant to the glycosidic O4 atoms are quite insensitive to the guest inclusion, and their values span certain ranges for an annular CD conformation, including (i) the glucose inclination angle (*τ*); (ii) the endocyclic torsion angles *φ* [O5(*n* + 1)–C1(*n* + 1)–O4(*n*)–C4(*n*)], *ψ* [C1(*n* + 1)–O4(*n*)–C4(*n*)–C5(*n*)], of which their sum of averages is close to null for a round CD structure [39]; (iii) the deviation distances of O4 atoms from their mean plane—the more close to zero values, the less distorted the CD; and (iv) the adjacent O4(*n*)···O4(*n* − 1), O4(*n*)···centroid distances—the less fluctuated values for a well-defined heptagon formed by seven O4 atoms (Table S2). Group 2: (v) the O3(*n*)···O2(*n* + 1) distances indicate the systematic intramolecular, interglucose H-bonds securing the CD round structure. These two parameter groups define the CD skeleton, of which their non-H atoms (C1–C6, O2–O5) are used for quantifying the CD similarity via the structure superposition. Group 3: (vi) the exocyclic torsion angles *χ* [C4–C5–C6–O6], *ω* [O5–C5–C6–O6] describe the freely rotating O6–H groups, of which their values are largely varied and hence excluded from the calculation of rms fits.

**Figure 2:**
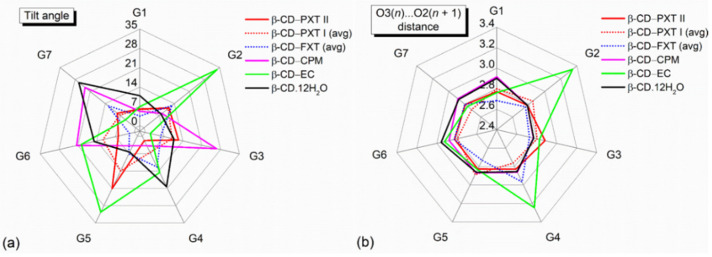
Radar plots of (**a**) tilt angles and (**b**) O3(*n*)∙∙∙O2(*n* + 1) distances of the β-CD glucose units (G1–G7) depicting the host conformational changes upon inclusion of the halogen-containing antidepressants, clomipramine (CPM) HCl [32], PXT HCl (form II), PXT base (form I [35]) and fluoxetine (FXT) HCl [34], of which the averages of two similar β-CD monomers in the β-CD dimer are shown. For comparison, data of the inclusion complex of β-CD–(–)-epicatechin (EC) [42] and the uncomplexed β-CD·12H_2_O [40] are included; see also Table S2. Angles and distances are in ° and Å.

**Figure 3:**
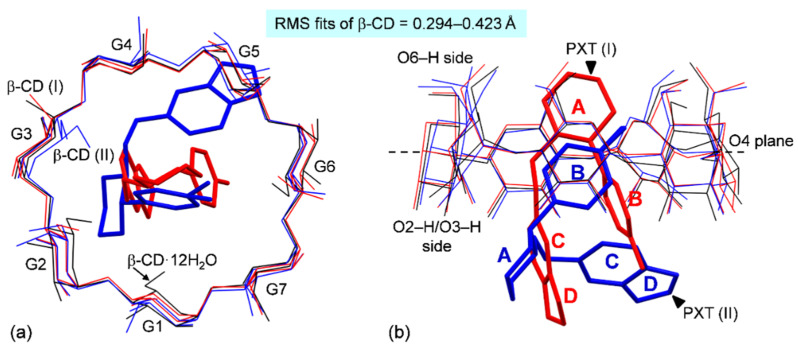
Structure overlays of the two β-CDs in complex with PXT (wireframes: blue—form II and red—form I (main host β-CD #2 [35]), and the uncomplexed β-CD·12H_2_O (black wireframe [40]), viewed from (**a**) the top and (**b**) the side. RMS fits are computed for the host β-CDs, excluding O6, H atoms and guests.

The microscopic structure comparison of the β-CD macrocycles of I (#2 [35]), II and β-CD·12H_2_O [40] shows that their elemental parameters are similar and fall in the normal ranges: (i) tilt angles *τ*, 1.8–26.2°; (ii) torsion angles *φ*, *ψ*, 103.0–120.0°, −95.9° to −130.3°; (iii) O4 deviations, −0.196 to 0.277 Å; (iv) adjacent O4 distances, O4···centroid distances, 4.242–4.548 Å, 4.794–5.327 Å; and (v) O3(*n*)···O2(*n* + 1) distances, 2.684–2.966 Å (Figure 2 and Table S2). To dig deeper into the seven composing glucose units, the puckering parameters *Q*, *θ* [41] are also similar and span in the short ranges of 0.544–0.583 Å, 1.0–7.7°, indicating that all the glucose units are in a normal chair conformation. The macroscopic structure comparison shows that the β-CD macrocycles of I (#2 [35]), II and β-CD·12H_2_O [40] are similarly round, as indicated by the rms fits of 0.294–0.423 Å (Figure 3).

The orientation of flexible O6–H groups with respect to the central cavity should be further noted. There are three doubly disordered O6–H groups of glucose residues 2, 3 and 7. Most of the O6–H groups (8/10) are pointed outward from the β-CD cavity to H-bond with neighboring OH groups and water sites (Table S3), as indicated by *χ*, *ω* of 45.3–68.0°, −53.8° to −76.6° (Table S2). Conversely, the twofold disordered C63–O63–H are pointed inward to the β-CD cavity with *χ*, *ω* having opposite signs of −160.3° to −176.7°, 65.4–68.4° (Table S2).

At this point, a question remained—at what circumstances of inclusion complexation does the CD macrocycle become significantly distorted from a round structure? An answer is depicted as the long spikes in the radar plots of the glucose tilt angles (Figure 2a) and the O3(*n*)···O2(*n* + 1) distances (Figure 2b). The parameters compared here are of β-CDs in complex with halogen-containing antidepressants with high efficacy in treating depression (e.g., TCAs—clomipramine (CPM) and SSRIs—fluoxetine (FXT) and PXT) and powerful polyphenol antioxidants. As TCAs/SSRIs of a nonpolar nature are mainly held in the β-CD cavity by weak intermolecular C/O–H···π interactions, the β-CD macrocycles are slightly affected by the drug inclusion [32,34]; their tilt angles and O3(*n*)···O2(*n* + 1) distances are similar to those of β-CD·12H_2_O [40], Figure 2a,b. On the contrary, tea (+)-catechin (CA) and (−)-epicatechin (EC) have their polyphenolic rings (A/B) deeply inserted in the β-CD cavity and are primarily stabilized by intermolecular O–H···O H-bonds [42]. The two diametrically opposed glucose units G2, G5 are strongly inclined >30°, resulting in the disruption of systematic O3(*n*)···O2(*n* + 1) H-bonds due to the separation distances of O32···O23 and O34···O25 > 3.2 Å and hence, β-CD is more distorted from a round structure [42]. Moreover, other similar examples include the inclusion complexes of β-CD·diclofenac sodium·11H_2_O [43] and α-CD·p-nitrophenol·3H_2_O and α-CD·p-hydroxybenzoic acid·3H_2_O [44].

### 2.2. Variation of Inclusion Scenarios of Conformationally Flexible PXT

**Figure 4:**
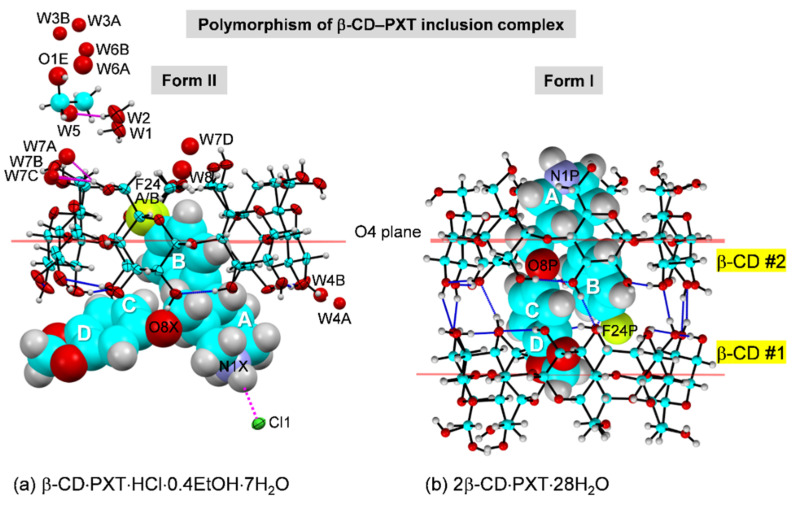
Distinct inclusion scenarios of (**a**) 1:1 β-CD–PXT HCl (form II) and (**b**) 2:1 β-CD–PXT base (form I [35]). ORTEP diagrams are drawn at a 30% probability level. For clarity, the PXT molecules are shown in the space-filling model and water molecules in form I are omitted. Note that PXT exists in freebase (form I [35]), whereas PXT is protonated as PXT–H^+^ and directly linked by Cl^−^ (form II), see magenta dotted line. The O–H∙∙∙O hydrogen bonds within β-CDs and between various molecules are shown with blue and magenta connecting lines, respectively.

PXT is the largest and least structurally rigid among the SSRIs as it comprises two aromatic rings (B and C), one non-aromatic six-membered ring (A), and one five-membered ring (D). Whereas the PXT A-rings of two crystal forms adopt a regular chair conformation, the D-rings of PXT (II) and PXT (I) exist in a twist and an envelope form, with corresponding puckering parameters *Q*, *φ* [41] of 0.103 Å, 164° and 0.219 Å, 325.7°, respectively (Table 1). PXT has two chiral centers at C3 and C4 on the A-ring possessing the (3*S*,4*R*) stereochemistry for the marketed pharmacological form (Scheme 1 and Figure 1). This facilitates the pointing up of the perpendicular B-ring to the horizontal plane and the pointing down of the -CH_2_–O- bridge linked to the C–D-rings, thus reducing steric repulsion between the two bulky groups; see the relevant torsion angles C5–C4–C18–C19 and C2–C3–C7–O8 in Table 1. The PXT conformations can be parameterized by two torsion angles *κ* [C4–C3–C7–O8] and *ε* [C3–C7–O8–C9]. For the polymorphic β-CD–PXT complex, the different *κ, ε* values of 167.7°, −83.4° and 87.2°, −131.7° define the two distinct PXT conformations, i.e., II, open, V-shaped; and I, closed, U-shaped (Figure 4). PXT molecules in distinct lattice circumstances that exist in different (*κ*, *ε*) coordinates on the PESs are compared in Section 2.5. Moreover, other parameters distinguishing the two PXT structures include the interplanar angles and the centroid–centroid distances between the B- and C-rings, which are 65.7°, 5.457 Å and 9.0°, 3.637 Å, for PXT (II) and PXT (I), respectively (Table 1). Different PXT conformations result in distinct inclusion scenarios, host–guest interactions, stoichiometric ratios (see below) and 3D arrangements (see Section 2.3).

A glimpse of Figure 4 gives an impression of the open V-shaped PXT partly embedded in the β-CD cavity, in contrast to the closed U-shaped PXT entirely encapsulated in the β-CD dimeric cavity, leading to further explorations of the complex thermodynamic stabilities as well as PXT conformational stabilities in respective Section 2.4 and Section 2.5. A gaze at Figure 4 reveals fine details of inclusion geometries. PXT (II) inserts the B-ring from the β-CD wider perimeter (O2–H/O3–H), giving a 1:1 host–guest complex (Figure 4a). Because PXT(II) is in an open V-shaped conformation, the B-ring is inclined 76.3° against the β-CD molecular plane, and the B-ring centroid is 1.107 Å beneath the O4 plane (Figure 4a). The shallow insertion of the PXT (II) B-ring in the β-CD cavity is to optimize interactions between the PXT (II) A-, C–D-rings with the β-CD O2–H/O3–H side. Hence, PXT (II) is maintained in position by the H-bond network of N1X–H1(···O61)···Cl1(···H–O66)···H2–O1W and O25–H···π(C) and C31–H···π(B) interactions (Table 2). The partly inclusion of PXT (B-ring) in the β-CD cavity allows the host molecules to pack in a channel mode; see Section 2.3. By contrast, PXT (I [35]) in a closed U-shaped structure is optimally embedded in the β-CD dimeric cavity, yielding a 2:1 host–guest complex. The A-ring is included in β-CD #2 such that its plane passing through C2–C3–C5–C6 makes an angle of 72.2° against the O4 plane, and its centroid is 2.029 Å above this plane (Figure 4b). The mostly parallel B- and C-rings are in the intermolecular interstices within the dimer, and the D-ring is enclosed in β-CD #1 (Figure 4b). PXT (I) is stabilized intramolecularly by face-to-face π(B)···π(C) interaction and is kept in position in the β-CD dimeric cavity by engaging intermolecularly in the O63_1–H···N1P–H···O53 H-bonds and a number of C–H···π(B,C) interactions (Table 2). β-CD dimers with a nearly complete inclusion of PXT are arranged in a screw-channel style; see Section 2.3.

A question raised at the end of the section for the β-CD–TCA/SSRI complexes, was what the meaning of the common inclusion modes of the aromatic rings bearing halogen atoms? Antidepressants carrying halogens on their aromatic rings have high efficacy in treating depression, e.g., CPM, FXT, PXT, and these rings are secured in the formed channel and cavity of β-CDs [32,34]. Particularly, PXT and FXT commonly contain an aryloxypropylamine portion and fluorine atoms and are encapsulated in the β-CD dimeric cavity [34]. The SSRIs have a specific binding to the SERT pocket via their halogen atoms [45,46].

### 2.3. Similar Channel-Type Packing through Different Host–Guest Ratios and Inclusion Modes

The crystallographic evidence of β-CD–SSRI inclusion complexes based on the β-CD dimeric motif anchoring two sertraline HCl, two fluoxetine HCl in the triclinic, space group *P*1 [34] and one PXT base in the monoclinic, space group *P*2_1_ [35] hinted to us that the 2:2 or 1:1 β-CD–PXT complex could plausibly exist. Crystallization trials ultimately yielded the 1:1 β-CD–PXT HCl inclusion complex in the triclinic, space group *P*1, the crystal form II, reflecting a rare phenomenon of polymorphism in CD inclusion complexes.

**Figure 5:**
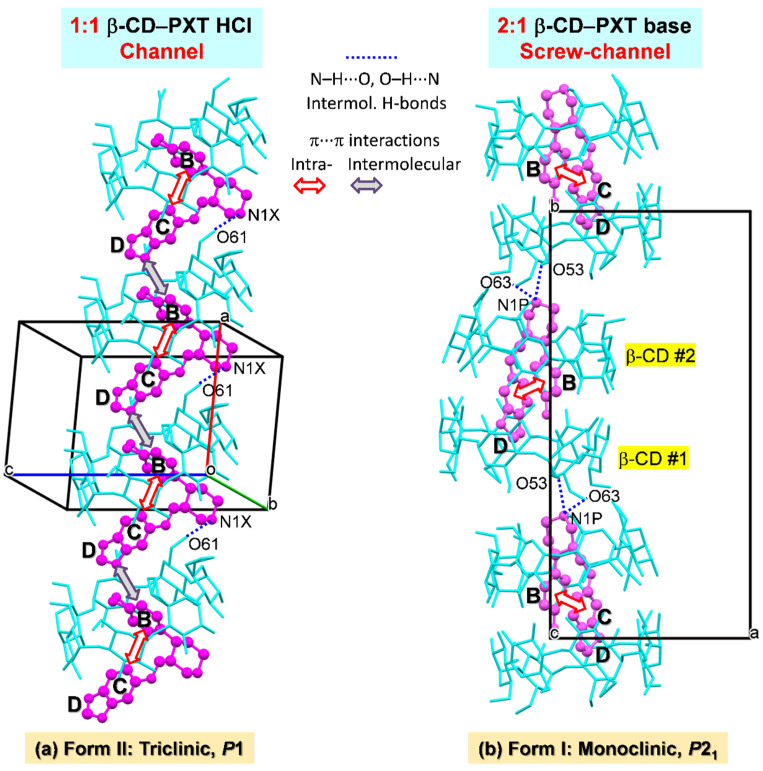
Elusive polymorphism of CD inclusion complexes demonstrated for (**a**) 1:1 β-CD–PXT HCl in triclinic, *P*1 (form II) packing in the channel structure and (**b**) 2:1 β-CD–PXT base in monoclinic, *P*2_1_ (form I) existing in the screw-channel structure [35]. β-CD macrocycles and PXT molecules are shown with cyan wireframes and magenta ball-and-stick models, respectively. For better visibility, water molecules, chlorides and H-atoms are omitted. The crystal lattices are stabilized not only by O–H∙∙∙O H-bonds within and between β-CDs, but also by the host–guest N–H···O, O–H···N H-bonds, intramolecular face-to-face π∙∙∙π (form I) and edge-to-face intra-, intermolecular π∙∙∙π and C–F···π interactions (form II).

PXT (II), in a more open V-shaped conformation, inserts its B-ring into the β-CD cavity and lays the A- and C–D-rings nearby the O2–H/O3–H side in the intermolecular spaces. β-CDs are packed along the *a*-axis to form a channel, which is maintained by host–guest N1X–H1∙∙∙O61 H-bond and guest–guest edge-to-face π(B)∙∙∙π(C), C–F∙∙∙π(D) interactions (Figure 5a and Table 2 and Table S3). Although the peculiar U-shaped structure of PXT in the uncomplexed form is unstable (Section 2.5), it is certainly stabilized by a total encapsulation in the β-CD dimeric cavity (Section 2.4). The dimeric motifs are stacked along the *b*-axis to form a screw channel, which is stabilized by the host–guest O63_1–H∙∙∙N1P–H∙∙∙O53 H-bonds between the twofold screw rotation symmetry-related dimers (Figure 5b and Table 2). Plus, we explain in Section 2.4 that the structure of the 2:1 β-CD–PXT complex has an optimum host–guest interactions than does the structure of the unimolar complex due to the greater presence of dispersion interactions. Section 2.5 further adds that the U-shaped structure of PXT in the β-CD dimeric cavity is the least stable conformation compared to PXT in the uncomplexed state and PXT in complex with SERT protein.

### 2.4. Thermodynamic Stabilities of the 1:1 and 2:1 β-CD–PXT Inclusion Complexes

**Figure 6:**
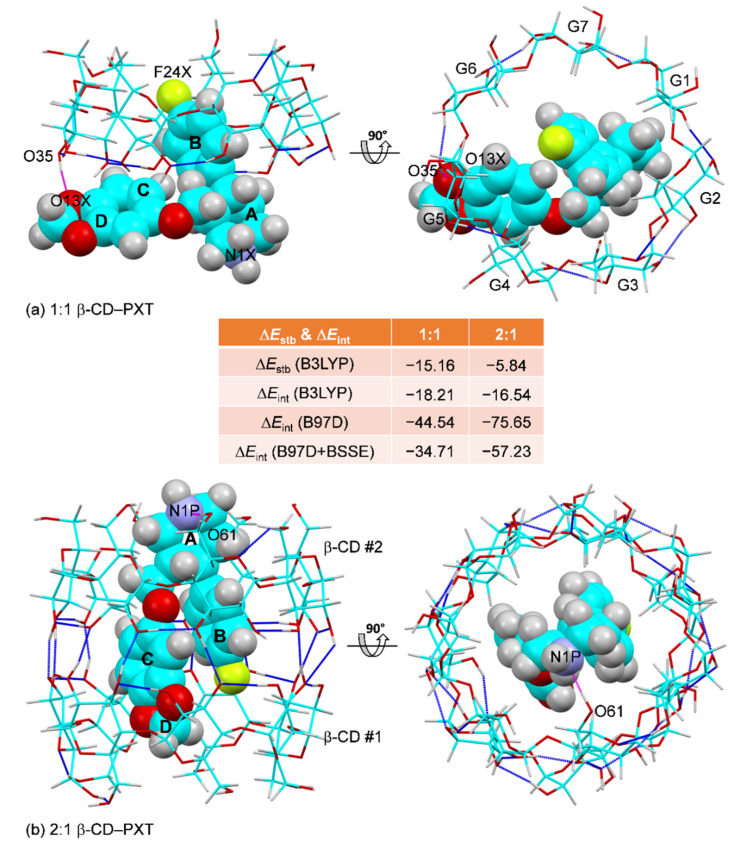
Inclusion complexes of (**a**) 1:1 β-CD−PXT base and (**b**) 2:1 β-CD−PXT base derived from DFT full-geometry optimization in the gas phase; side view (left) and top view (right). Values of stabilization energy and interaction energy (Δ*E*_stb_ and Δ*E*_int_) are given in the inset; see also Table 2 and Tables S4–S6. The O–H∙∙∙O H-bonds within β-CD and host–guest O–H∙∙∙O/N H-bonds are indicated by blue and magenta connecting lines, respectively. The estimated uncertainties of Δ*E* = 0.1 kcal mol^−1^.

Principally, the three nonpolar structural moieties of PXT (piperidine—A-ring; 4-fluorophenyl—B-ring; 1,3-benzodioxole—C–D-rings) can be competitively bound to the β-CD cavity, yielding a trimodal β-CD–PXT inclusion complex. In solution, NMR combined with molecular dynamics revealed that the 1:1 β-CD–PXT base is quite stable with a binding constant of ~2000 M^−1^, and the C–D-ring moiety is deeply inserted into the β-CD cavity [37]. This inclusion mode agrees with the NMR data of the β-CD encapsulation of the 1,3-benzodioxole moiety of berberine HCl (a plant extract with numerous health benefits) even though the binding constant based on phase solubility is somewhat small, 101.78 M^−1^ [47]. We envisage that upon slow solvent evaporation of concentrated β-CD–PXT solution during crystallization, PXT prefers to insert its shorter fragment, either the A-ring or B-ring, from the wider side (O2–H/O3–H) into the β-CD cavity, yielding a stable, more compact β-CD–PXT inclusion complex. Then the complex molecules form clusters, aggregates, stable nuclei, and ultimately single crystals of the β-CD–PXT inclusion complex with different host–guest ratios: 2:1 (form I in the monoclinic, space group *P*2_1_); or 1:1 (form II in the triclinic, space group *P*1), Figure 6. Therefore, to gain a meaningful structure–energy relationship of the polymorphic β-CD–PXT inclusion complex, we took the corresponding X-ray structures for energy minimization by DFT calculation.

Weak intermolecular interactions are vital in the thermodynamic stabilization of supramolecular CD inclusion complexes. Apart from H-bond interactions, the dispersion forces play an important role in non-covalent host–guest complexation. Moreover, supramolecules contain a large number of atomic orbitals; thus, an error due to the superposition of basis sets needs to be considered, as demonstrated in our previous work on the β-CD–TCA inclusion complexes [33]. Both β-CD–PXT stoichiometric complexes are stabilized by similar host–guest interaction schemes, i.e., O35–H···O13X(D) H-bond, O25–H···π(C), C31–H···π(B) interactions for the 1:1 complex and O61_2–H···N1P(A) H-bond, C36_1–H···π(C), C36_2–H···π(B) interactions for the 2:1 complex (Figure 6 and Table 2 and Table S4). Considering the molecular deformation and host–guest interactions, the resulting stabilization energies (Δ*E*_stb_s) of −15.16 and −5.84 kcal mol^−1^ indicate that the 1:1 complex is ~2.5 times more energetically favorable than the 2:1 complex (Table S5). However, when both host and guest are considered as rigid molecules (the constituents of the fully optimized structure of a complex), the interaction energies (Δ*E*_int_s) of both complexes are more similar even though the order of values retains −18.21 kcal mol^−1^ (1:1) vs. −16.54 kcal mol^−1^ (2:1), Table S5. Further inclusions of the dispersion interactions (based on B97D functional) and the BSSE correction, the resulted Δ*E*_int_s of the complexes are −44.54, −34.71 kcal mol^−1^ (1:1) and −75.65, −57.23 kcal mol^−1^ (2:1). This suggests that the β-CD dimer anchors a U-shaped PXT molecule with the corrected Δ*E*_int_s ~1.7 times more stable than the β-CD single molecule, which accommodates a V-shaped PXT (Table S6). Note that the magnitudes of BSSE contributing to the dispersion-corrected Δ*E*_int_s are 22% and 24% for the 1:1 and 2:1 β-CD–PXT complexes, respectively (Table S6), in agreement with those of the β-CD–TCA complexes [33].

### 2.5. Rationale of PXT Conformational Flexibility through Potential Energy Surfaces (PESs)

**Figure 7:**
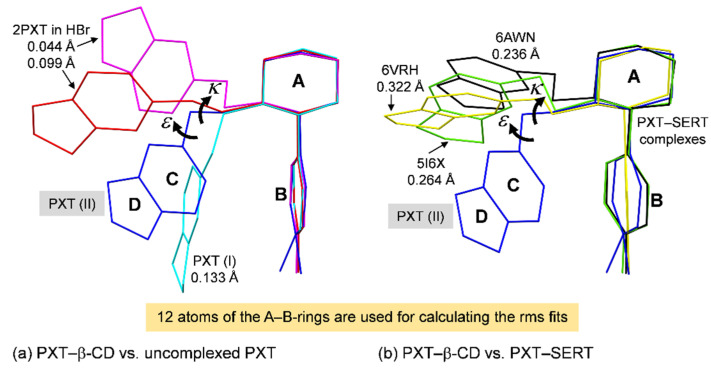
Structure overlays of PXT in different lattice environments, comparing PXT embedded in the β-CD cavity with (**a**) the free HBr salt form and (**b**) PXT bound to the SERT pocket. For the brief descriptions of the PXT–SERT complexes, see footnotes h–j of Table 3. PXT (II) as a reference structure is indicated by blue sticks, of which their names are marked in gray areas, and only rigid moieties of PXT [N1–C6 & C18–C23] are used for calculating the rms fits (see atom numbering in Scheme 1). The corresponding rms fit for each structure pair is indicated by a nearby distance. Torsion angles *κ*, *ε* responsible for the PXT conformational flexibility are marked.

Among SSRIs, PXT, with the largest and most diverse structure, exhibits the highest conformational flexibility, as evidenced by the structure overlay in our recent work [34]. Particularly, the C–D-rings are connected at the chiral center C3 via the rather freely rotating -CH_2_–O- bridge, and their rotation with respect to the A-ring is controlled by torsion angles *κ*, *ε* (Figure 7a,b). This inspires us with the plausibility of PXT to exist in other more open conformations rather than the closed U-shaped structure entrapped in the β-CD dimeric cavity (form I [35]). Hence, crystallization attempts were made and were finally fruitful, as described in Section 3.1.

Scrutinizing the varied PXT structures allows us to get to the bottom of their conformational flexibility. The distinction of crystal lattices covers PXT in an uncomplexed state, PXT entrapped in the carrier (CD) cavity and PXT in complex with SERT protein. Whereas the A-rings are in a similar chair conformation, the planar B-rings linked at C4 are more-or-less perpendicular to the plane passing through the C2–C3–C5–C6 atoms. This is indicated by torsion angle C5–C4–C18–C19 in a short span of −119.6° to −130.5° for the uncomplexed PXT and PXT (I, II), and in a larger range of −94.1° to −154.3° for PXT–SERT complexes (Table 3). This is reflected from the corresponding respective rms fits of 0.044–0.133 Å and 0.236–0.322 Å, which are computed from the rather rigid A–B-rings and those of PXT (II), which is a reference structure (Figure 7a,b). If the C–D-rings are also included (i.e., all atoms are considered) for the calculation, the rms fits exceed 3 Å. Although a glimpse of the C–D-rings shows similar planar structures in varied circumstances, a gaze at them reveals that the D-rings mostly adopt an envelope form, except for those of PXT (II) in a rare twist form and PXT–SERT (6AWN) in a planar structure (Figure 7a,b). This is because 1,3-dioxole has a large out-of-plane thermal motion, giving rise to its existence as either a puckered form or a planar structure with a small energy barrier of 0.36 kcal mol^−1^ [48]. PXT molecules in the varied lattice environments, of which the C-rings mostly make acute-to-right angles (42.3–87.6°) with the B-rings and the centroid–centroid distances, are 5.457–7.197 Å (namely, the open V- to L-shaped conformations). Conversely, PXT is conformationally forced to exist in a closed U-shaped conformation in the β-CD dimeric cavity with an interplanar angle of the B and C ring of 9.0° and the centroid–centroid distance of 3.637 Å (Figure 7a,b and Table 3).

**Figure 8:**
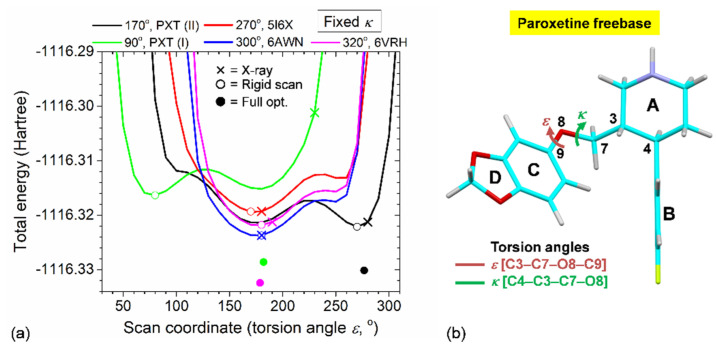
(**a**) PESs of PXT freebase derived from a rigid scan of torsion angle *ε* at different fixed torsion angle *κ*, mimicking PESs in three distinct lattice environments, i.e., PXT in an uncomplexed state with two molecules in the asymmetric unit (*κ* = 180°, 300°), PXT in complex with β-CD (form II, *κ* = 170°; form I, *κ* = 90°) and PXT in complex with SERT (5I6X, *κ* = 270°; 6AWN, *κ* = 300°; 6VRH, *κ* = 320°). Note that 6VRH is obtained from cryo-EM; see text for more details. (**b**) Initial atomic coordinates for the rigid scan are taken from the PXT–β-CD complex (form II), showing two key torsion angles *κ*, *ε* that define the PXT conformational flexibility. The coordinates (*κ*, *ε*) of PXT from X-ray analysis (✕), rigid scan (○—*E*_tot_min_) and full optimization (●—*E*_opt_) by DFT calculations are marked. Note that *E*_opt_s of the three PXT–SERT complexes are coincident; see Figure 9 for the fully optimized PXT structures and Table 3 for the summary.

The PXT structural flexibility plays a pivotal role in governing its pharmacological functions. The distinct conformations of PXT arising from two torsion angles *κ* [C4–C3–C7–O8] and *ε* [C3–C7–O8–C9] in various lattice circumstances, of which their crystal data can be retrieved from two large crystallographic databases, including the Cambridge Crystallographic Data Center (CCDC; www.ccdc.cam.ac.uk [49] accessed on 14 November 2021) for small molecules and the RCSB Protein Data Bank (RCSB PDB; www.rcsb.org [50] accessed on 16 October 2021) for macromolecules. The coordinates (*κ*, *ε*) of the relevant X-ray structures after rounding for PES scans are classified into three groups as follows (see × and ○ in Table 3 and Figure 8). (1) PXT in an uncomplexed state with two molecules in the asymmetric unit (180°, 170°; 300°, 180°) (code TUZFIF [51]). (2) PXT in the β-CD (carrier) cavity (170°, 280° (form II, this work); 90°, 230° (form I, code BEGWEQ [35]). (3) PXT in action while in the bound state to the central sites of three SERT proteins (270°, 180° (code 5I6X [52]); 300°, 180° (code 6AWN [38]); 320°, 190° (code 6VRH), which is determined by cryo-electron microscopy (cryo-EM) [46]). Whereas 5I6X has the wild-type SERT [52], 6AWN and 6VRH have the S439T mutant of SERT and SERT together with the recombinant Fab 8B6, respectively [38,46]. To rationalize thermodynamic stabilities of the seven PXT conformers—whether and to what extent the gas-phase structures are different from their solid-state structures, we performed the DFT calculations in two consecutive steps for economic computing in the vast PES: (i) the PES rigid scans of five distinct PXT conformers, each with fixed *κ* and varied *ε* (30–310°), and (ii) the complete-geometry optimization of the five structures with the minimum total energy from the PES scans to obtain the conformer at global minimum energy.

**Figure 9:**
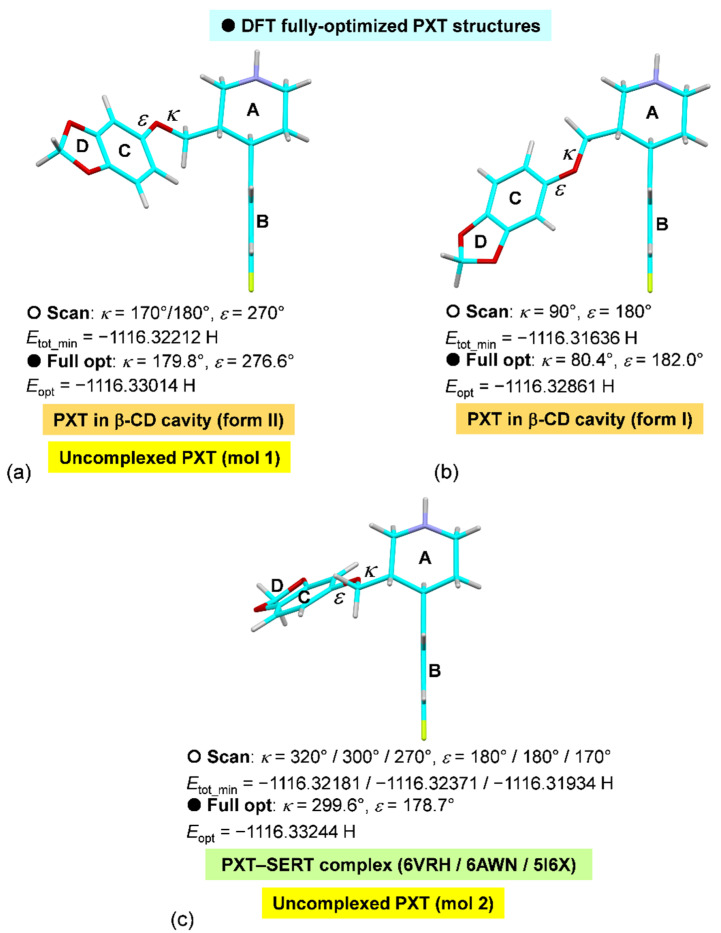
Fully optimized PXT structures from DFT calculation in the gas phase. The starting structures are taken from the PES rigid scan of PXT in three varied crystal lattices, including (**a**,**b**) PXT encapsulated in the β-CD cavity and (**c**) PXT bound to the central site of SERT. Note that the uncomplexed PXT comprises two molecules in the asymmetric unit, of which their torsion angles *κ* and *ε* are mostly identical with PXT (II) and PXT (6AWN); see text for more details. The coordinates (*κ*, *ε*) of PXT from PES rigid scan (○) and full optimization (●) together with *E*_tot_min_ and *E*_opt_ from DFT calculations are given; see Figure 8 for the PESs of PXT and Table 3 for the summary.

Figure 8 depicts the PESs based on total energy (*E*_tot_) of five PXT conformers with fixed *κ* = 90°, 170°, 270°, 300° and 320° and scan coordinate *ε* = 30–310° with an increment of 10°. After the PES rigid scans, to obtain a minimum total energy (*E*_tot_min_) for each conformer (see ○ in Table 3 and Figure 9), PXT (II) had an *E*_tot_min_ at the coordinate (*κ*, ε) = (170°, 270°), while those of the other four PXT conformers were at (90°, 80°) for PXT (I), (270°, 170°) for 5I6X, (300°, 180°) for 6AWN and (320°, 180°) for 6VRH. Clearly, all five PESs have a common feature of one saddle and two wells with the total energy differences and energy barriers of <1 and ~3 kcal mol^−1^ for PXT (II) partially and PXT (I) totally embedded in the β-CD cavity, and of ~4 and ~4 kcal mol^−1^ for PXT in complex with SERT (5I6X, 6AWN and 6VRH), Figure 8. This indicates that one conformer (coincident with the X-ray structure) is energetically preferable over the other one. For the uncomplexed PXT, HBr comprises two different molecules in the asymmetric unit [51], whereas PXT molecule 1 is similar to PXT–β-CD form II, PXT molecule 2 is like PXT–SERT (6AWN), Figure 9 and Table 3.

The PXT structures with *E*_tot_min_ from the PES scans including PXT–β-CD form II (ε = 270°) and the rest (ε = 80°–180°) are subsequently used for full-geometry optimization. The energy minimization provided genuine global minimums of the completely optimized structures, which are lower than *E*_tot_min_ of the structures from constrained PESs. The corresponding *κ*, ε, *E*_opt_ are 179.8°, 276.6°, −1116.33014 Hartree (PXT–β-CD form II, PXT HBr molecule 1); 80.4°, 182.0°, −1116.32861 Hartree (PXT–2β-CD form I); and 299.6°, 178.7°, −1116.33244 Hartree (PXT–SERT—5I6X, 6AWN, 6VRZ and PXT HBr molecule 2).

To sum up, we can infer from the PES profiles depicted in Figure 8 as follows: (i) PXT (II) at (*κ*, ε) = (170°, 270°) is the most stable and is 3.16 kcal mol^−1^ more stable than PXT (I), which is the least stable with rather high *E*_tot_min_ at (*κ*, ε) = (90°, 80°) (Figure 9a,b). This implies that the unstable U-shaped structure of PXT (I) is stabilized when encapsulated in the β-CD cavity. (ii) PXT in the HBr salt form comprises two thermodynamically stable molecules, which are equivalent to PXT (II) and PXT–SERT. This is evidenced by the coincident (*κ*, ε) coordinates of the X-ray, PES scanned and fully optimized structures (Figure 9a,c). (iii) From PES scans, the X-ray structures of PXT–SERT (5I6X, 6AWN and 6VRH) are the more to most energetically stable conformers (Figure 9c). After energy minimization, the three optimized structures of PXT in complex with SERT are identical with the lowest *E*_opt_, suggesting the reliability of the calculation results obtained. Note that the energetically favorable conformation of PXT in the SERT binding pocket indicates that PXT itself is stable and induces the conformational changes of the surrounding amino acids for optimal binding interactions and antidepressant function [36].

## 3. Materials and Methods

### 3.1. Materials

PXT HCl hemihydrate (≥98%) was obtained from Acros Chemicals (code 462630). β-CD (≥95%) was purchased from Cyclolab, Budapest, Hungary (code CY-2001). Absolute EtOH (≥99.8%) was supplied by Liquor Distillery Organization, Excise Department, Chachoengsao, Thailand. All chemicals were used as received. The ultrapure water was obtained from a Milli-Q Water System.

### 3.2. X-ray Crystallography

#### 3.2.1. Single-Crystal Preparation

Several attempts were made to reproduce colorless, prismatic single crystals of the 2:1 β-CD–PXT base inclusion complex in the monoclinic, space group *P*2_1_ (form I), which were prepared by kneading of the solid mixture, dissolving in pure water at 313 K and solvent evaporation at 293 K [35]. Here, we used a crystallization method of slow solvent evaporation as in our previous works, yielding the 1:1 β-CD–TCA HCl [33] and the 2:2 β-CD–SSRI(sertraline/fluoxetine) HCl [34]. Note that the more water-soluble drugs in the HCl form gave both monomeric and dimeric β-CD complexes in the single-crystalline state. By contrast, the less water-soluble drugs in freebase, like PXT, co-crystallized with β-CD in the stoichiometric ratio of 1:2 [35]. Moreover, the independent crystallization attempts via solvent evaporation of the aqueous solutions of β-CD–amitriptyline HCl [30] and β-CD–amitriptyline base [53] yielded mostly isomorphous crystal structure and HCl salt found in the intermolecular interstices had a marginal effect on the complex structure [30].

β-CD 50 mg (0.044 mmol) and PXT HCl·0.5H_2_O 17 mg (0.022 mmol) were dissolved in 750 mL of 50% (*v*/*v*) EtOH–H_2_O at 323 K, giving a concentrated complex solution. A vial containing the complex solution was left to stand still in an air-conditioned lab room at 298 K. After a month of slow solvent evaporation (during the work from home period due to the 2nd wave of the COVID-19 pandemic in Bangkok in January 2021), it was ultimately fruitful to obtain colorless, thick plate-like crystals. Surprisingly, preliminary X-ray analysis revealed a new crystal form, i.e., form II of the 1:1 β-CD–PXT HCl inclusion complex in the triclinic space group *P*1.

#### 3.2.2. X-ray Diffraction Experiment

Different pieces of thick plate-like single crystals were selected, cut and separately mounted in a thin-walled glass capillary (Hilgenberg, Germany). They were screened for consistent unit cell constants and sufficient diffracting power. All crystals belonged to the triclinic system with similar unit cell parameters, suggesting a new crystal form of the β-CD–PXT complex. A cut rod-shaped crystal (0.32 × 0.34 × 0.44 mm) with the best diffracting power was chosen for X-ray data collection at 296 K to 0.70-Å atomic resolution on a Bruker APEXII CCD area-detector diffractometer (MoKα radiation; λ = 0.71073 Å). Data processing was carried out according to standard procedures using the APEX2 software suite [54]: (i) integration with SAINT [55], (ii) scaling and multi-scan absorption correction with SADABS [54] and (iii) merging with XPREP [55]. The data statistics, including the numbers of collected/unique/observed reflections, completeness and *R*_int_ values were 67,271/23,459/14,844, 99.8% and 0.0296, respectively.

#### 3.2.3. Structure Solution and Refinement

The crystal structure of the β-CD–PXT complex in the triclinic system (form II) was solved by the intrinsic phasing method with SHELXTL XT [54], providing all non-H atoms of β-CD and PXT molecules with full site occupation, hence the host–guest stoichiometric ratio was 1:1. The missing non-H atoms of solvents (water and ethanol) and chloride ions were located by difference Fourier electron density maps graphically assisted by WinCoot [56]. The protonated PXT molecule (at N1X axial position, H2) was counterbalanced and directly coordinated (at N1X equatorial position, H1) by a fully occupied chloride (Figure 1), as normally observed in the crystal structures of TCAs and SSRIs in free HCl salt form. By contrast, most crystal structures of the β-CD encapsulation of TCAs and SSRIs had two half-occupied chlorides, which were not in direct contact with the protonated drugs, as pointed out in our previous works [33,34].

The disorders of β-CD O6–H groups and solvents caused trouble in the refinement. There were 2–3 doubly disordered C6–H_2_–O6–H groups (G2, G3 and G7) in β-CD and 5 of 7 water molecules distributed over 12 positions (site occupancy factors (SOFs) = 0.3–0.6) in the intermolecular interstices; 2 water molecules were fully occupied. Plus, the peak cluster was better modeled as the disordered ethanol site (SOF = 0.4). All non-H atoms were refined anisotropically by full-matrix least-squares on *F*^2^ using SHELXTL XLMP [54], except for the disordered solvents, which were refined isotropically. H atom positions of rigid groups were placed geometrically and treated with a riding model: C−H = 0.93 Å, *U*_iso_ = 1.2*U*_eq_(C)(aromatic); C−H = 0.98 Å, *U*_iso_ = 1.2*U*_eq_(C)(methine); C−H = 0.97 Å, *U*_iso_ = 1.2*U*_eq_(C)(methylene); C−H = 0.96 Å, *U*_iso_ = 1.5*U*_eq_(C)(methyl); and N−H = 0.89 Å, *U*_iso_ = 1.2*U*_eq_(2° ammonium). The H-atoms of β-CD OH groups and two fully occupied water molecules, O1W and O2W, were initially located by difference Fourier maps; those of disordered water sites could not be found. Then, the OH H-atoms were refined using ‘AFIX 147’ or ‘AFIX 83’ with restraints O−H = 0.84 Å, *U*_iso_ = 1.5*U*_eq_(O). The water H-atoms were refined with DFIX restraints to idealized geometry (O−H 0.96 Å and H∙∙∙H 1.52 Å) and with ‘AFIX 30’ constraint *U*_iso_ = 1.5*U*_eq_(water). Moreover, to prevent short H∙∙∙H distances in the refinement, BUMP antibumping restraints were applied. The refinement converged to a final *R*_1_ = 0.0633. For more details of data collection and refinement statistics, see Supplementary Materials, Table S1.

The crystal structure refinement of the 2:1 β-CD–PXT base (form I) with a final *R*_1_ = 0.0662 deserved further notes for comparison [35]. Due to the limited number of diffraction data and quality, all β-CD C-atoms and several tens of disordered water sites were refined isotropically, whereas the β-CD O-atoms, PXT most non-H atoms and 13 fully occupied water molecules were refined anisotropically. Moreover, 1 out of 14 O6–H groups of the head-to-head β-CD dimer was found to be doubly disordered.

### 3.3. DFT Calculations

#### 3.3.1. Full-Geometry Optimization of 1:1 and 2:1 β-CD-PXT Complexes

Both host CD and guest drug exhibit conformational flexibility to an extent, particularly in this case, where the primary hydroxyls O6–H of β-CD and the CH_2_–O group bridge the A- and C–D-rings of PXT. This is to attain thermodynamically favorable CD inclusion complexes with optimum host–guest interactions, reflecting the importance of the induced-fit mechanism [57]. Hence, an arbitrary starting structure of a CD inclusion complex could plausibly lead to a local minimum in the potential energy surface, giving rise to a meaningless energy-optimized structure. On the other hand, the meaningful structure–energy relationship can be reliably established via complete-geometry optimization of the initial X-ray-derived structure using density functional theory (DFT) calculation. This has been successfully demonstrated in our previous works on tea catechins [58], olive polyphenols [59], coffee chlorogenic acid [60] for the H-bond-stabilized complexes, and antidepressant drugs including TCAs [33], SSRIs [34] for the CH···π-stabilized complexes.

Thus far, there have been two crystal modifications of 2β-CD·PXT base·28H_2_O (monoclinic, *P*2_1_—form I [35]) and β-CD·PXT HCl·0.4EtOH·7H_2_O (triclinic, *P*1—form II; this work), indicating the polymorphism in the β-CD encapsulation of PXT. To evaluate the host–guest interactions in the two crystal forms, we considered the 2:1 and 1:1 β-CD–PXT base for the respective forms I and II without water of hydration. The corresponding atomic coordinates of both phases from X-ray analysis, of which the underestimated X–H bond distances were corrected to neutron hydrogen distances: C–H, 1.083 Å; N–H, 1.009 Å; and O–H, 0.983 Å [61]. The normalized structures were firstly optimized by the semiempirical PM3 method and subsequently fully re-optimized by DFT calculation using the B3LYP functional in the gas phase with mixed basis sets 6-31+G* for H, N, O and 4-31G for C. All calculations were carried out using program GAUSSIAN09 [62] on a DELL PowerEdge T430 server. Stabilization energy and interaction energy (Δ*E*_stb_ and Δ*E*_int_) of the 2:1 and 1:1 host–guest complexes were calculated using Equations (1) and (2).

Δ*E*_stb_ = *E*_cpx_ − (*E*_mβ-CD_opt_ + *E*_D_opt_) (1)

Δ*E*_int_ = *E*_cpx_ − (*E*_mβ-CD_sp_ + *E*_D_sp_) (2)

where *E*_cpx_, *E*_mβ-CD_opt_ and *E*_D_opt_ are the molecular energies from the full-geometry optimization of complex, monomeric (m = 1)/dimeric (m = 2) host β-CD and drug PXT, respectively; *E*_mβ-CD_sp_ and *E*_D_sp_ are the corresponding single-point energies in the complexed states.

#### 3.3.2. Dispersion and BSSE Corrections of Interaction Energies

In the vicinity of weak intermolecular interactions, such as CH···π type, which primarily stabilize β-CD inclusion complexes with TCAs [32,33] and SSRIs [34], the dispersion forces play an important role in the derivation of more reasonable estimates of thermodynamic stabilization. Plus, the supramolecular CD inclusion complexes with large numbers of ghost orbitals (basis functions without electrons or protons) significantly affect the host–guest interaction energies. Therefore, to improve the DFT results, two approximations, including the dispersion corrected functional B97D and the basis set superposition error (BSSE), based on the counterpoise method [63], were further considered. We calculated single-point energies Δ*E*_int_s with the dispersion-correction (B97D) and the BSSE correction of the structures optimized at the B3LYP/6-31+G*/4-31G level.

#### 3.3.3. Construction of the Potential Energy Surfaces (PESs) of PXT Conformers

A thorough conformational comparison in our previous work [34] and the distinct PXT structures in the inclusion complexes β-CD–PXT forms I and II hinted to us a further exploration of the conformation–total energy relationship, that is, the potential energy surface (PES). We categorized the PXT conformations into three groups, reflecting their existences and functions, which covered the uncomplexed PXT, PXT in the CD (carrier) cavity and PXT in action, while in the bound state to the SERT pocket (Table 3). Among SSRI drugs, PXT had the most diverse structural components, including piperidine (A-ring) linked with 4-fluorophenyl (B-ring) on C4 and connected to 1,3-benzodioxole (C–D-rings) via bridging the -CH_2_–O- group on C3. The B-ring had a more or less perpendicular torsion angle with respect to the plane passing through C2–C3–C5–C6 of the A-ring, as found in the β-CD–PXT form II, which was used for the PES rigid scans.

The PXT conformational flexibility between the A- and C–D-rings was described by two torsion angles C3–C7–O8–C9 and C4–C3–C7–O8, namely *ε* and *κ*, respectively (Figure 1). Inspecting the PXT structures revealed that torsion angles *κ*, *ε* are (see ✕ in Table 3): 179.7°, 173.4° and −60.0°, −176.3° for the respective molecules 1 and 2 of PXT HBr·0.5H_2_O (code TUZFIF [51]); 87.2°, −131.7° for the 2:1 β-CD–PXT (code BEGWEQ, form I [35]); 167.7°, −83.4° for the 1:1 β-CD–PXT (form II, this work); −91.1°, −177.1° for the SERT–PXT complex (code 5I6X [52]); −55.7°, −176.2° (code 6AWN [38]); and −36.9°, −172.0° (code 6VRH [46]). To perform the PES rigid scans at the B3LYP/6-31+G* level in the gas phase, we fixed *κ* at (see ○ in Table 3): (i) 90° for PXT in form I [35]; (ii) 170° for PXT in form II and PXT HBr molecule 1 [51]; (iii) 300° for PXT HBr molecule 2 [51] and PXT in 6AWN [38]; (iv) 270° for PXT in 5I6X [52]; and (v) 320° for PXT in 6VRH [46]. At each fixed *κ* angle, the *ε* angles were rigid scanned in the range of 30–310° with a 10°-step. Then, the global minimums of (i)–(v) were fine-tuned for exact torsion angles *κ*, *ε* (genuine minimums; see ● in Table 3) by full-geometry optimization at the B3LYP/6-31+G* level in the gas phase.

## 4. Conclusions

Our study series aims at forwarding insights on the β-cyclodextrin (β-CD)–selective serotonin reuptake inhibitor (SSRI) inclusion complexes by X-ray crystallography combined with DFT calculation. This work focuses on the polymorphism of β-CD–paroxetine (PXT) complex and the distinctive nature of PXT conformational flexibility, which are inspired by the reported 2:1 β-CD–PXT complex (crystal form I [35]).

Here, we add the 1:1 β-CD–PXT complex to the list of elusive polymorphisms in CD inclusion complexes. Crystal form II of β-CD·PXT HCl·0.4EtOH·7H_2_O belongs to the triclinic, space group *P*1, whereas crystal form I of the 2β-CD·PXT base·28H_2_O pertains to the monoclinic, space group *P*2_1_ [35]. The β-CD–PXT polymorphism stems from the PXT conformational flexibility, which is defined by torsion angles *κ*, *ε* around the bridge -CH_2_–O- connecting the A- and C–D-rings. While PXT (II) in an open V-shaped conformation has the B-ring shallowly inserted into the β-CD cavity, PXT (I) in a closed U-shaped structure is mostly entirely embedded in the β-CD dimeric cavity; the A-ring is deeply inserted in the main β-CD cavity. Even though the distinct PXT inclusion scenarios and conformations are notable, PXT molecules in both crystal forms are similarly maintained in the CD cavity via host–guest N–H···O5/O6 H-bonds and C/O–H···π(B/C) interactions and β-CDs have similar 3D arrangements, channel (II) vs. screw-channel (I).

Further theoretical explorations on the β-CD–PXT thermodynamic stabilities and the PXT conformational stabilities based on their potential energy surfaces (PESs) have been completed by DFT calculations. The complex interaction energies (Δ*E*_int_s) with corrections of the dispersion and the basis set superposition error (BSSE) indicate that the 2:1 β-CD–PXT complex with the greater presence of dispersion interactions is more energetically favorable than the unimolar complex. Conversely, PXT molecules existing in varied lattice environments, including the uncomplexed PXT HBr salt, PXT embedded in the β-CD cavity (forms I and II) and PXT in action while bound at the central site of the serotonin transporter (SERT) protein, suggest the contrary. Whereas free PXT, PXT (II) and PXT–SERT complex are more energetically stable, PXT (I) is the least stable. Here, the PXT conformational flexibility and stabilities are comprehensively investigated for the first time, suggesting the true nature of this pivotal SSRI drug. The coronavirus disease 2019 (COVID-19) pandemic caused havoc on both economics and physical and mental health. It seemed not to end by mass vaccination. As there is a growing body of evidence that SSRIs could lessen the COVID-19 severity [6,7,8,9,10], the CD inclusion complexation not only helps to improve the drug bioavailability, but also promotes the use of antidepressants and COVID-19 medicines concurrently [5].

## Figures and Tables

**Table 1:** Comparisons of geometrical parameters and inclusion structures of PXT in the 1:1 β-CD–PXT HCl (crystal form II) and the 2:1 β-CD–PXT base (crystal form I).

Polymorphism of β-CD–PXT complex	PXT (II)	PXT (I) ^a^
(1) Geometrical parameters		
D-ring puckering ^b^		
*Q* (Å)	0.103(15)	0.219
*φ* (°)	164(8)	325.7
Conformation	Twist	Envelope
B vs. C interplanar angle (°)	65.7(2)	9.0
Centroid–centroid distance (Å)		
A–B	4.282	4.347
A–C	5.580	6.100
B–C	5.457	3.637
Selected torsion angles (°) ^c^		
C5–C4–C18–C19	−129.7(4)	−120.5
C2–C3–C7–O8	−68.3(6)	−150.4
C7–O8–C9–C10	3.1(6)	−9.9
C4–C3–C7–O8, *κ*	167.7(4)	87.2
C3–C7–O8–C9, *ε*	−83.4(6)	−131.7
(2) Inclusion structure		
Ring embedded in CD cavity ^d^	B	A
Interplanar angle (°)		
A-ring vs. β-CD O4 plane	50.2(2) ^g^	72.2 ^g^
B-ring vs. β-CD O4 plane	76.3(1)	69.3
C-ring vs. β-CD O4 plane	39.0(2)	78.3
Distance from drug to β-CD (Å)		
A/B-ring centroid to O4 centroid (diagonal) ^e^	−1.107	2.029
A/B-ring centroid to O4 plane (vertical)	−0.871	1.640
Chiral center C4X/C4P to O4 centroid (diagonal) ^f^	−3.733	−0.931

^a^ PXT base embedded in the β-CD dimeric cavity [35]. All the piperidine A-rings of PXT molecules exist as a normal chair form in different lattice circumstances; hence, the 1,3-dioxole D-ring parameters are shown. ^b^ 5-membered D-ring puckering coordinates, including radius *Q* and meridian angle *φ* [41]. ^c^ For atom numbering, see Scheme 1. ^d^ For the 2:1 β-CD–PXT base complex (form I), the main host of β-CD #2 is considered [35]. ^e^ When the β-CD O6-side pointing upwards, the positive(negative) values indicate that the A/B-ring centroid of a drug molecule is above(beneath) the β-CD O4 plane; see Figure 4. ^f^ Chiral center C4 is considered, see Scheme 1. ^g^ Plane passing through C2–C3–C5–C6 of the piperidine A-ring.

**Table 2:** Selected intermolecular interactions stabilizing the 1:1 and 2:1 β-CD–PXT inclusion complexes as derived from X-ray analysis and DFT calculation. ^a^

Interaction	D–H	H···A	D···A	∠(DHA)	Interaction	D–H	H···A	D···A	∠(DHA)
X-ray									
1:1 β-CD–PXT HCl					2:1 β-CD–PXT base				
(form II)					(form I) ^d^				
N1X–H1···O61 ^i b^	0.89	1.95	2.792(6)	158.1	O63_1–H···N1P ^iii b^	0.99	1.863	2.846	169.3
N1X–H1···Cl1	0.89	2.38	3.131(5)	141.6	N1P–H···O53_1 ^iv^	1.02	2.534	3.172	120.5
O66–H···Cl1 ^ii^	0.82	2.29	3.084(5)	163.4	C36_1–H···*Cg*3(B) ^e^	1.00	3.628	4.519	149.6
O1W–H2···Cl1 ^ii^	0.96	2.28	3.154(5)	151.0	C37_1–H···*Cg*2(C)	1.00	3.685	4.641	160.6
O25–H···*Cg*2(C) ^c^	0.82	3.410	4.228	176.0	C35_2–H···*Cg*3(B)	1.00	3.717	4.620	151.5
C31–H···*Cg*3(B)	0.98	3.623	4.548	158.2	C36_2–H···*Cg*3(B)	1.00	3.262	4.248	168.4
DFT ^f^									
1:1 β-CD–PXT base					2:1 β-CD–PXT base				
O35–H···O13X	0.98	1.97	2.92	162.3	O61_2–H···N1P	0.99	1.91	2.90	172.8
O25–H···*Cg*2(C) ^c^	0.98	3.74	4.34	121.8	C36_1–H···*Cg*2(C) ^c^	1.10	3.57	4.65	168.3
C31–H···*Cg*3(B)	1.10	3.66	4.74	165.3	C36_2–H···*Cg*3(B)	1.10	3.50	4.55	161.1

^a^ For complete list of intermolecular interactions, see Supplementary Materials, Table S3 (X-ray) and Table S4 (DFT). ^b^ Equivalent positions: (i) *x* − 1, *y*, *z*; (ii) *x* + 1, *y*, *z* + 1 [form II: triclinic, *P*1]; (iii) −*x* + 2, y − 0.5, −*z* + 2; (iv) −*x* + 2, y + 0.5, −*z* + 2 [form I: monoclinic, *P*2_1_]. ^c^ PXT HCl: *Cg*2 = C-ring (C9X–C10X–C11X–C12X–C16X–C17X), *Cg*3 = B-ring (C18X–C19X–C20X–C21X–C22X–C23X). ^d^ For the 2:1 β-CD–PXT base inclusion complex (form I [35]), two β-CDs are additionally numbered 1 and 2. ^e^ PXT base: *Cg*2 = C-ring (C9P–C10P–C11P–C12P–C16P–C17P), *Cg*3 = B-ring (C18P–C19P–C20P–C21P–C22P–C23P). ^f^ DFT full-geometry optimization in vacuum at the B3LYP/6–31+G*/4–31G level using X-ray structures of the 1:1 β-CD–PXT (form II) and 2:1 β-CD–PXT (form I) with PXT in the neutral form as starting models.

**Table 3:** Conformational parameters of PXT in uncomplexed form, in β-CD cavity (I, II) and in complex with SERT, derived from X-ray analysis and DFT calculation.

X-ray	Free HBr Form		In β-CD Cavity		SERT–PXT Complex		
	Mol 1 ^f^	Mol 2 ^f^	PXT (I) ^g^	PXT (II)	5I6X ^h^	6AWN ^i^	6VRH ^j^
Molecular shape	L	L	U	V	L	L	L
Ring-D puckering ^a^							
*Q* (Å)	0.198	0.246	0.219	0.103(15)	0.193	-	0.097
*φ* (°)	144.5	141.6	325.7	164(8)	144.4	-	215.9
Conformation	Env.	Env.	Env.	Twist	Env.	Planar	Env.
B vs. C interplanar angle (°)	75.3	42.3	9.0	65.7(2)	58.3	56.0	87.6
Centroid–centroid dist. (Å)							
A–B	4.313	4.279	4.347	4.282	4.318	4.312	4.284
A–C	6.342	6.042	6.100	5.580	6.175	6.139	6.070
B–C	7.197	6.341	3.637	5.457	6.018	6.161	6.067
Selected torsion angles (°)							
C5–C4–C18–C19	−119.6	−130.5	−120.5	−129.7(4)	−154.3	−150.7	−94.1
C2–C3–C7–O8	−58.2	64.8	−150.4	−68.3(6)	30.4	64.8	85.7
C7–O8–C9–C10	−147.4	−40.9	−9.9	3.1(6)	102.8	64.5	−139.8
✕ C4–C3–C7–O8, *κ* ^b^	179.7	−60.0	87.2	167.7(4)	−91.1	−55.7	−36.9
✕ C3–C7–O8–C9, *ε* ^b^	173.4	−176.3	−131.7	−83.4(6)	−177.1	−176.2	−172.0
RMS fit (Å) ^c^	0.099	0.044	0.133	0	0.264	0.236	0.322
DFT							
(i) PES rigid scan							
○ *κ* (fixed) ^b^	180	300	90	170	270	300	320
○ *ε*_min_ (scanned 30–310°) ^d^	270	180	80	270	170	180	180
*E*_tot_min_ (Hartree) ^e^	−0.31681	−0.32371	−0.31636	−0.32212	−0.31934	−0.32371	−0.32181
(ii) Full optimization							
● *κ* ^b^	179.8	299.6	80.4	179.8	299.6	299.6	299.6
● *ε* ^b^	276.6	178.7	182.0	276.6	178.7	178.7	178.7
*E*_opt_ (Hartree) ^e^	−0.33014	−0.33244	−0.32861	−0.33014	−0.33244	−0.33244	−0.33244

^a^ (3*S*,4*R*)-PXT has two non-planar six-membered A-ring and five-membered D-ring. Since all the A-rings in various lattice environments adopt a normal chair conformation, the D-ring puckering coordinates, including radius *Q* and meridian angle *φ* of the envelope (env.) and twist forms are given [41]. ^b^ The coordinates (*κ*, *ε*) of PXT from X-ray analysis (✕), rigid scan (○) and full optimization (●) by DFT calculations are marked; see Figure 8 for the PESs and Figure 9 for the fully optimized structures and Scheme 1 for atom numbering. ^c^ RMS fits are computed from the rigid moieties of PXT [N1–C6 and C18–C23], and PXT (II) is a reference structure. ^d^ Torsion angle *ε* at the global minimum (*ε*_min_) of PES rigid scan. ^e^ Minimum total energy (*E*_tot_min_) from PES scan and total energy from energy minimization (*E*_opt_) in Hartree (H); only the decimal values after −1116 H are given. ^f^ PXT HBr·0.5H_2_O with two molecules per asymmetric unit (CSD code: TUZFIF) [51]. ^g^ PXT in the β-CD dimeric cavity (CSD code: BEGWEQ) [35]. ^h^ PXT in complex with SERT at the central site, of which its structure was determined by X-ray crystallography at 3.14 Å resolution (PDB code: 5I6X) [52]. ^i^ PXT in complex with the serine to threonine at position 439 (S439T) mutant of SERT at the central site, of which its structure was determined by X-ray crystallography at 3.62 Å resolution (PDB code: 6AWN) [38]. ^j^ PXT in complex with SERT and the recombinant antibody fragment (Fab) 8B6, of which its structure was determined by cryo-EM at 3.30 Å resolution (PDB code: 6VRH); 6AWN as initial model [46].

## Data Availability

The data are contained within the article. Additional crystallographic and computational data are available in Supplementary Materials and the Cambridge Crystallographic Data Centre (CCDC).

## Supplementary Materials

The following are available online at https://www.mdpi.com/article/10.3390/ph15010098/s1, Table S1: X-ray single-crystal data collection and refinement statistics of β-CD–PXT HCl (form II); Table S2: Selected geometrical parameters of β-CD–PXT HCl (form II); Table S3: Hydrogen bond parameters and π···π interactions in β-CD⋅PXT⋅HCl⋅0.4EtOH⋅7H_2_O; Table S4: Hydrogen bond parameters in 1:1 and 2:1 β-CD–PXT inclusion complexes from DFT full-geometry optimization; Table S5: Stabilization and interaction energies of 1:1 and 2:1 β-CD–PXT inclusion complexes from DFT full-geometry optimization; Table S6: Dispersion- and BSSE-corrected interaction energies of 1:1 and 2:1 β-CD–PXT inclusion complexes from DFT/B97D calculations. Crystallographic data of II have been deposited at the Cambridge Crystallographic Data Centre (CCDC) under reference number 2115511.

## References

[1] World Health Organization (WHO) (2021). Depression. https://www.who.int/news-room/fact-sheets/detail/depression.

[2] Wang Y., Kala M.P., Jafar T.H. (2020). Factors associated with psychological distress during the coronavirus disease 2019 (COVID-19) pandemic on the predominantly general population: A systematic review and meta-analysis. PLoS ONE.

[3] World Health Organization (WHO) Director-General’s Opening Remarks at the Media Briefing on COVID-19—3 March 2020. https://www.who.int/director-general/speeches/detail/who-director-general-s-opening-remarks-at-the-media-briefing-on-covid-19---3-march-2020.

[4] Worldometers COVID Live Update. 7 November 2021. https://www.worldometers.info/coronavirus/.

[5] Mohebbi N., Talebi A., Moghadamnia M., Taloki Z.N., Shakiba A. (2020). Drug interactions of psychiatric and COVID-19 medications. Basic Clin. Neurosci..

[6] Lenze E.J., Mattar C., Zorumski C.F., Stevens A., Schweiger J., Nicol G.E., Reiersen A.M. (2020). Fluvoxamine vs placebo and clinical deterioration in outpatients with symptomatic COVID-19: A randomized clinical trial. JAMA.

[7] Hoertel N., Sánchez-Rico M., Vernet R., Beeker N., Jannot A.S., Neuraz A., Limosin F. (2021). Association between antidepressant use and reduced risk of intubation or death in hospitalized patients with COVID-19: Results from an observational study. Mol. Psychiatry.

[8] Meikle C.K.S., Creeden J.F., McCullumsmith C., Worth R.G. (2021). SSRIs: Applications in inflammatory lung disease and implications for COVID-19. Neuropsychopharmacol. Rep..

[9] Reis G., dos Santos Moreira-Silva E.A., Silva D.C.M., Thabane L., Milagres A.C., Ferreira T.S., Mills E.J. (2021). Effect of early treatment with fluvoxamine on risk of emergency care and hospitalisation among patients with COVID-19: The TOGETHER randomised, platform clinical trial. Lancet Glob. Health.

[10] Schloer S., Brunotte L., Mecate-Zambrano A., Zheng S., Tang J., Ludwig S., Rescher U. (2021). Drug synergy of combinatory treatment with remdesivir and the repurposed drugs fluoxetine and itraconazole effectively impairs SARS-CoV-2 infection in vitro. Br. J. Pharmacol..

[11] Food and Drug Administration (FDA) (2019). Depression Medicines. https://www.fda.gov/consumers/free-publications-women/depression-medicines.

[12] Uekama K., Irie T., Otagiri M., Hoshino T., Yamada Y., Ohtani Y. (1983). Protective effects of cyclodextrins on the haemolysis induced with imipramine in vitro. Membrane.

[13] 13. Hoshino T., Hirayama F., Uekama K., Yamasaki M. (1989). Reduction of protriptyline-photosensitized hemolysis by β-cyclodextrin complexations. Int. J. Pharm..

[14] Passos J.J., de Sousa F.B., Lula I.S., Barreto E.A., Lopes J.F., de Almeida W.B., Sinisterra R.D. (2011). Multi-equilibrium system based on sertraline and β-cyclodextrin supramolecular complex in aqueous solution. Int. J. Pharm..

[15] Passos J.J., de Sousa F.B., Mundim I.M., Bonfim R.R., Melo R., Viana A.F., Sinisterra R.D. (2012). In vivo evaluation of the highly soluble oral β-cyclodextrin–Sertraline supramolecular complexes. Int. J. Pharm..

[16] Buko V., Palecz B., Belica-Pacha S., Zavodnik I. (2017). The Supramolecular Complex of Sertraline with Cyclodextrins: Physicochemical and Pharmacological Properties. Nano-and Microscale Drug Delivery Systems.

[17] Abouhussein D.M., el Nabarawi M.A., Shalaby S.H., El-Bary A.A. (2021). Sertraline-Cyclodextrin Complex Orodispersible Sublingual Tablet: Optimization, Stability, and Pharmacokinetics. J. Pharm. Innov..

[18] Morin-Crini N., Fourmentin S., Fenyvesi É., Lichtfouse E., Torri G., Fourmentin M., Crini G. (2021). 130 years of cyclodextrin discovery for health, food, agriculture, and the industry: A review. Environ. Chem. Lett..

[19] Adeoye O., Cabral-Marques H. (2017). Cyclodextrin nanosystems in oral drug delivery: A mini review. Int. J. Pharm..

[20] Matencio A., Navarro-Orcajada S., García-Carmona F., López-Nicolás J.M. (2020). Applications of cyclodextrins in food science. A review. Trends Food Sci. Technol..

[21] Bakshi P.R., Londhe V.Y. (2020). Widespread applications of host-guest interactive cyclodextrin functionalized polymer nanocomposites: Its meta-analysis and review. Carbohydr. Polym..

[22] Fourmentin S., Crini G., Lichtfouse E. (2018). Cyclodextrin Applications in Medicine, Food, Environment and Liquid Crystals.

[23] Diniz T.C., Pinto T.C.C., Menezes P.D.P., Silva J.C., Teles R.B.D.A., Ximenes R.C.C., Almeida J.R.G.D.S. (2018). Cyclodextrins improving the physicochemical and pharmacological properties of antidepressant drugs: A patent review. Expert Opin. Ther. Pat..

[24] Karpinski P.H. (2006). Polymorphism of active pharmaceutical ingredients. Chem. Eng. Technol..

[25] Aree T., Chaichit N., Engkakul C. (2008). Polymorphism in β-cyclodextrin–benzoic acid inclusion complex: A kinetically controlled crystal growth according to the Ostwald’s rule. Carbohydr. Res..

[26] Fernandes J.A., Ramos A.I., Ribeiro-Claro P., Paz F.A.A., Braga S.S. (2015). Studies on polymorph conversion in a new cyclodextrin inclusion compound. CrystEngComm.

[27] Censi R., di Martino P. (2015). Polymorph impact on the bioavailability and stability of poorly soluble drugs. Molecules.

[28] Hilfiker R., Von Raumer M. (2019). Polymorphism in the Pharmaceutical Industry: Solid Form and Drug Development.

[29] Bernstein J. (2020). Polymorphism in Molecular Crystals 2e. International Union of Crystallography.

[30] Aree T. (2020). β-Cyclodextrin encapsulation of nortriptyline HCl and amitriptyline HCl: Molecular insights from single-crystal X-ray diffraction and DFT calculation. Int. J. Pharm..

[31] Aree T. (2020). β-Cyclodextrin inclusion complexation with tricyclic antidepressants desipramine and imipramine: A structural chemistry perspective. J. Pharm. Sci..

[32] Aree T. (2020). Supramolecular Complexes of β-Cyclodextrin with Clomipramine and Doxepin: Effect of the Ring Substituent and Component of Drugs on Their Inclusion Topologies and Structural Flexibility. Pharmaceuticals.

[33] Aree T. (2021). Distinctive Supramolecular Features of β-Cyclodextrin Inclusion Complexes with Antidepressants Protriptyline and Maprotiline: A Comprehensive Structural Investigation. Pharmaceuticals.

[34] Aree T. (2021). Advancing insights on β-cyclodextrin inclusion complexes with SSRIs through lens of X-ray diffraction and DFT calculation. Int. J. Pharm..

[35] Caira M.R., de Vries E., Nassimbeni L.R., Jacewicz V.W. (2003). Inclusion of the antidepressant paroxetine in β-cyclodextrin. J. Incl. Phenom. Macrocycl. Chem..

[36] Tavoulari S., Forrest L.R., Rudnick G. (2009). Fluoxetine (Prozac) binding to serotonin transporter is modulated by chloride and conformational changes. J. Neurosci..

[37] Bernini A., Spiga O., Ciutti A., Scarselli M., Bottoni G., Mascagni P., Niccolai N. (2004). NMR studies of the inclusion complex between β-cyclodextrin and paroxetine. Eur. J. Pharm. Sci..

[38] Coleman J.A., Gouaux E. (2018). Structural basis for recognition of diverse antidepressants by the human serotonin transporter. Nat. Struct. Mol. Biol..

[39] French A.D., Johnson G.P. (2007). Linkage and pyranosyl ring twisting in cyclodextrins. Carbohydr. Res..

[40] Lindner K., Saenger W. (1982). Crystal and molecular structure of cyclohepta-amylose dodecahydrate. Carbohydr. Res..

[41] Cremer D.T., Pople J.A. (1975). General definition of ring puckering coordinates. J. Am. Chem. Soc..

[42] Aree T., Jongrungruangchok S. (2016). Crystallographic evidence for β-cyclodextrin inclusion complexation facilitating the improvement of antioxidant activity of tea (+)-catechin and (−)-epicatechin. Carbohydr. Polym..

[43] Caira M.R., Griffith V.J., Nassimbeni L.R., van Oudtshoorn B. (1994). Synthesis and X-ray crystal structure of β-cyclodextrin diclofenac sodium undecahydrate, a β-CD complex with a unique crystal packing arrangement. J. Chem. Soc. Chem. Commun..

[44] Harata K. (1977). The structure of the cyclodextrin complex. V. Crystal structures of α-cyclodextrin complexes with p-nitrophenol and p-hydroxybenzoic acid. Bull. Chem. Soc. Jpn..

[45] Zhou Z., Zhen J., Karpowich N.K., Law C.J., Reith M.E., Wang D.N. (2009). Antidepressant specificity of serotonin transporter suggested by three LeuT–SSRI structures. Nat. Struct. Mol. Biol..

[46] Coleman J.A., Navratna V., Antermite D., Yang D., Bull J.A., Gouaux E. (2020). Chemical and structural investigation of the paroxetine-human serotonin transporter complex. eLife.

[47] Zhang Y., Cui Y.L., Gao L.N., Jiang H.L. (2013). Effects of β-cyclodextrin on the intestinal absorption of berberine hydrochloride, a P-glycoprotein substrate. Int. J. Biol. Macromol..

[48] Emanuele E., Orlandi G. (2005). Large amplitude out-of-plane vibrations of 1,3-benzodioxole in the S_0_ and S_1_ states: An analysis of fluorescence and excitation spectra by ab initio calculations. J. Phys. Chem..

[49] Groom C.R., Bruno I.J., Lightfoot M.P., Ward S.C. (2016). The Cambridge structural database. Acta Cryst..

[50] Rose P.W., Prlić A., Bi C., Bluhm W.F., Christie C.H., Dutta S., Young J. (2015). The RCSB Protein Data Bank: Views of structural biology for basic and applied research and education. Nucleic Acids Res..

[51] Carvalho P.S., de Melo C., Ayala A.P., da Silva C.C., Ellena J. (2016). Reversible solid-state hydration/dehydration of paroxetine HBr hemihydrate: Structural and thermochemical studies. Cryst. Growth Des..

[52] Coleman J.A., Green E.M., Gouaux E. (2016). X-ray structures and mechanism of the human serotonin transporter. Nature.

[53] Castiglione F., Ganazzoli F., Malpezzi L., Mele A., Panzeri W., Raffaini G. (2017). Inclusion complexes of β-cyclodextrin with tricyclic drugs: An X-ray diffraction, NMR and molecular dynamics study. Beilstein J. Org. Chem..

[54] Bruker (2014). APEX2, SADABS and SHELXTL.

[55] Bruker (2008). SAINT and XPREP.

[56] Emsley P., Lohkamp B., Scott W., Cowtan K. (2010). Features and development of Coot. Acta Cryst..

[57] Koshland D.E. (1973). Protein shape and biological control. Sci. Am..

[58] Aree T., Jongrungruangchok S. (2016). Enhancement of antioxidant activity of green tea epicatechins in β-cyclodextrin cavity: Single-crystal X-ray analysis, DFT calculation and DPPH assay. Carbohydr. Polym..

[59] Aree T., Jongrungruangchok S. (2018). Structure—Antioxidant activity relationship of β-cyclodextrin inclusion complexes with olive tyrosol, hydroxytyrosol and oleuropein: Deep insights from X-ray analysis, DFT calculation and DPPH assay. Carbohydr. Polym..

[60] Aree T. (2019). Understanding structures and thermodynamics of β-cyclodextrin encapsulation of chlorogenic, caffeic and quinic acids: Implications for enriching antioxidant capacity and masking bitterness in coffee. Food Chem..

[61] Allen F.H., Bruno I.J. (2010). Bond lengths in organic and metal-organic compounds revisited: X—H bond lengths from neutron diffraction data. Acta Cryst..

[62] Frisch M.J.E.A., Trucks G.W., Schlegel H.B., Scuseria G.E., Robb M.A., Cheeseman J.R., Nakatsuji H. (2009). Gaussian 09, revision A.01.

[63] Boys S.F., Bernardi F.J.M.P. (1970). The calculation of small molecular interactions by the differences of separate total energies. Some procedures with reduced errors. Mol. Phys..

