# Supplementary material for: Inclusion Scenarios and Conformational Flexibility of the SSRI Paroxetine as Perceived from Polymorphism of β-Cyclodextrin–Paroxetine Complex
Source: Pharmaceuticals (Basel). 2022 Jan 14;15(1):98. doi: 10.3390/ph15010098 (PMC8781563; doi:10.3390/ph15010098)
Supplement: Supplementary file 1 [file pharmaceuticals-15-00098-s001.zip › pharmaceuticals-1526251-supplementary.pdf]

Supplementary Materials

Inclusion Scenarios and Conformational Flexibility of the SSRI Paroxetine as Perceived from Polymorphism of β-Cyclodextrin–Paroxetine Complex

Thammarat Aree

Department of Chemistry, Faculty of Science, Chulalongkorn University, Bangkok 10330, Thailand; athammar@chula.ac.th; ORCID: 0000-0002-7298-7401; Tel.: +66-2-2187584; Fax: +66-2-2187598

Supplementary materials available:

I. Crystallographic data

Table S1. X-ray single crystal data collection and refinement statistics of β-CD–PXT HCl (form II).

Table S2. Selected geometrical parameters of β-CD–PXT HCl (form II).

Table S3. Hydrogen bond parameters and π···π interactions in β-CD·PXT·HCl·0.4EtOH·7H_2O.

II. Computational data

Table S4. Hydrogen bond parameters in 1:1 and 2:1 β-CD–PXT inclusion complexes from DFT full-geometry optimization.

Table S5. Stabilization and interaction energies of 1:1 and 2:1 β-CD–PXT inclusion complexes from DFT full-geometry optimization.

Table S6. Dispersion- and BSSE-corrected interaction energies of 1:1 and 2:1 β-CD–PXT inclusion complexes from DFT/B97D calculations.

III. References

I. Crystallographic data

Table S1. X-ray single crystal data collection and refinement statistics of β-CD–PXT HCl (form II).

	β-CD–Paroxetine HCl
Abbreviated formula	β-CD·PXT·HCl·0.4EtOH·7H_2O
Chemical formula	$(C_6H_{10}O_5)_7 \cdot C_{19}H_{20}FNO_3 \cdot HCl \cdot 0.4(C_2H_6O) \cdot 7H_2O$
Formula weight	1635.25
Crystal habit, color	Thick plate, colorless
Crystal size [mm]	0.32 × 0.34 × 0.44
Crystal system, space group	Triclinic, *P*1 (No. 1)
a, *b*, *c* [Å]	11.4958(3), 12.3750(4), 15.0433(5)
α, *β*, γ [°]	111.979(1), 95.325(1), 100.104(1)
V [Å^3]	1924.09(10)
Z	1
D_c [g cm^{-3}]	1.411
μ [mm^{-1}]	0.156
F(000)	864
Diffractometer	APEXII Kappa CCD (Bruker)
Wavelength [Å]	MoK_α, 0.71073
T [K]	296(2)
Data collection	ω–ϕ scan, 1.0° step, 8 s expose
Frames collected	1376
θ range [°]	2.14–30.53
Resolution [Å]	0.70
Completeness [%], R_{int}	99.8, 0.0296
Reflns collected / unique / observed	67270 / 23458 / 14844
Data / parameters / restraints	23458, 1007, 31
R_1 [a], wR_2 [$I > 2\sigma(I)$] [b]	0.0633, 0.1582
R_1, wR_2 [all data], GoF	0.1061, 0.1854, 1.017
$\Delta\rho_{min}$, $\Delta\rho_{max}$ [e Å^{-3}]	–0.42, 0.48
Flack parameter (*x*)	0.03(2)
CCDC number	2115511

[a,b] $R = \sum||F_o| - |F_c||/\sum|F_o|$; $wR = \sum\{w(F_o^2 - F_c^2)^2/\sum w(F_o^2)^2\}^{1/2}$.

Table S2. Selected geometrical parameters of β-CD–PXT HCl (form II).

Residue n	Q [Å] [a] θ [°] [a]	τ [°] [b]	ϕ [c], ψ [c] [°]	O4 dev. [Å] [e]	O4 dist. [Å] [f]	O3O2 dist. [Å] [h]	χ [i], ω [i] [°]
1	0.572(8)	6.0(2)	110.4(4)	0.098(2)	4.467(4)	2.684(5)	56.9(4)
	3.0(8)		–116.6(4)		4.991(2)		–63.3(4)
2	0.555(9)	11.5(2)	116.5(3)	0.078(2)	4.313(3)	2.759(5)	58.1(7) [j] 52.9(12) [k]
	2.2(9)		–112.6(4)		5.066(2)		–70.2(7) [j] –60.2(11) [k]
3	0.559(9)	12.4(2)	113.3(3)	–0.144(2)	4.416(4)	2.727(5)	–176.7(7) [m] –160.3(13) [n]
	1.3(9)		–112.5(4)		5.059(3)		68.4(10) [m] 65.4(20) [n]
4	0.546(9)	1.8(3)	106.8(4)	–0.026(3)	4.338(5)	2.966(6)	58.4(5)
	3.0(9)		–124.2(4)		5.054(3)		–63.3(4)
5	0.550(9)	21.1(2)	116.2(4)	0.183(2)	4.395(4)	2.731(5)	68.0(5)
	4.8(9)		–97.7(5)		4.974(3)		–53.8(5)
6	0.572(9)	6.0(1)	109.9(4)	–0.092(2)	4.446(4)	2.826(6)	63.3(5)
	3.9(9)		–130.3(4)		5.136(3)		–57.3(5)
7	0.544(9)	8.4(2)	111.4(4)	–0.097(2)	4.293(4)	2.799(5)	45.3(9) [p] 63.7(11) [q]
	4.1(9)		–110.7(4)		5.038(2)		–76.6(8) [p] –58.2(11) [q]
			112.1 [d]		*0.174* [g]		
			–114.9 [d]		*0.162* [g]		
			–2.8 [d]		*0.868* [g]		

[a] Puckering parameters of an ideal cyclohexane chair (for R(C–C) = 1.54 Å) has puckering amplitude Q = 0.63 Å and angle describing the polar position $\theta = 0°$ [1].

[b] Tilt angle — interplanar angle of the plane through C1(n), C4(n), O4(n) and O4(n – 1) against the O4 plane.

[c] Endocyclic torsion angles ϕ and ψ at glycosidic O4, defined as O5(n + 1)–C1(n + 1)–O4(n)–C4(n) and C1(n + 1)–O4(n)–C4(n)–C5(n), respectively.

[d] Averages of ϕ and ψ are in *italics*; for the β-CD roundness, the sum of averages should be zero [2].

[e] Deviation of glycosidic O4 atoms from the least-squares plane through the seven O4 atoms.

[f] O4(n)···O4(n – 1) and O4(n)···centroid distances.

[g] Ranges of the O4(n)···O4(n – 1), O4(n)···centroid distances and the average of their ratios are in *italics*; for an ideal heptagon, the ratio is 0.868.

[h] O3(n)···O2(n + 1) distance

[i] Exocyclic torsion angles χ and ω are defined as C4–C5–C6–O6 and O5–C5–C6–O6, respectively.

[j,k] Twofold disordered C62–H_2–O62–H group with occupancy factors 0.61 and 0.39 for respective sites A and B.

[m,n] Twofold disordered C63–H_2–O63–H group with occupancy factors 0.64 and 0.36 for respective sites A and B.

[p,q] Twofold disordered O67–H group with occupancy factors 0.59 and 0.41 for respective sites A and B.

Table S3. (a) Hydrogen bond parameters in β-CD·PXT·HCl·0.4EtOH·7H_2O [Å, °].

D–H···A	D–H	H···A	D···A	∠(DHA)	D–H···A	D–H	H···A	D···A	∠(DHA)
β-CD–β-CD					O64–H···O7WA	0.82	1.80	2.59(2)	164.5
O21–H···O37	0.82	2.14	2.799(5)	137.3	O64–H···O7WB	0.82	1.95	2.728(11)	157.2
O31–H···O22	0.82	1.88	2.684(5)	167.5	O64–H···O7WC	0.82	2.09	2.864(16)	156.6
O61–H···O64 [i a]	0.82	1.97	2.775(5)	168.7	O26–H···O1W [viii]	0.82	1.90	2.698(6)	163.0
O22–H···O56 [iii]	0.82	1.97	2.788(4)	175.7	O36–H···O7WA [ix]	0.82	2.42	2.93(2)	121.2
O32–H···O23	0.82	1.99	2.759(5)	156.7	O66–H···Cl1 [x]	0.82	2.29	3.084(5)	163.4
O33–H···O24	0.82	1.92	2.726(5)	167.4	O37–H···O2W [ix]	0.82	2.04	2.756(7)	144.9
O63A–H···O31 [iv b]	0.82	1.81	2.597(8)	161.2	O67A–H···O3WA [ix]	0.82	1.91	2.730(18)	172.8
O34–H···O25	0.82	2.23	2.966(6)	148.7	O67B–H···O3WB [ix]	0.82	2.48	3.09(3)	132.8
O35–H···O26	0.82	1.91	2.731(5)	176.7	O1W–H1···O33 [xi]	0.96	2.15	2.956(6)	140.3
O65–H···O32 [vii]	0.82	2.06	2.831(5)	155.6	O2W–H1···O25 [ii]	0.96	1.96	2.827(7)	148.9
O36–H···O27	0.82	2.11	2.826(6)	145.5	*PXT–β-CD/H_2O/Cl*				
O27–H···O65 [ix]	0.82	1.95	2.766(5)	176.2	N1X–H1···O61 [viii]	0.89	1.95	2.792(6)	158.1
β-CD–H_2O/Cl				137.3	N1X–H1···Cl1	0.89	2.38	3.131(5)	141.6
O62A–H···O4WA [iv c]	0.82	2.17	2.825(11)	137.0	N1X–H1···O6WA [iii]	0.89	2.57	3.16(3)	124.9
O23–H···O3WA [v]	0.82	1.96	2.771(9)	173.2	O25–H···*Cg*2(C) [d]	0.82	3.410	4.228	176.0
O23–H···O3WB [v]	0.82	2.50	3.250(13)	152.0	C31–H···*Cg*3(B) [d]	0.98	3.623	4.548	158.2
O63B–H···O4WB [iv]	0.82	2.21	2.815(18)	130.8	*H_2O–/H_2O/Cl*				
O24–H···O4WA [vi]	0.82	2.14	2.794(7)	137.1	O1W–H2···Cl1 [x]	0.96	2.28	3.154(5)	151.0
O24–H···O4WB [vi]	0.82	1.96	2.700(13)	150.7	O2W–H2···O5W	0.96	1.84	2.74(3)	155.6

[a] Equivalent positions: (i) *x* + 1, *y* + 1, *z*; (ii) *x* + 1, *y*, *z*; (iii) *x*, *y*, *z* – 1; (iv) *x*, *y* – 1, *z*; (v) *x* – 1, *y*, *z* – 1; (vi) *x* – 1, *y* – 1, *z*; (vii) *x*, *y*, *z* + 1; (viii) *x* – 1, *y*, *z*; (ix) *x*, *y* + 1, *z*; (x) *x* + 1, *y*, *z* + 1; (xi) *x* + 1, *y* + 1, *z* + 1.

[b] Twofold disordered C62–H_2–O62–H group of β-CD with occupancy factors 0.61 and 0.39 for respective sites A and B.
Twofold disordered C63–H_2–O63–H group of β-CD with occupancy factors 0.64 and 0.36 for respective sites A and B.
Twofold disordered O67–H group of β-CD with occupancy factors 0.59 and 0.41 for respective sites A and B.

[c] Site occupancy factors (SOFs) are as follows:
7 water molecules are distributed over 14 sites: 1.0 (O1W, O2W); 0.6 (O3WA, O4WA, O7WD);
0.4 (O3WB, O4WB, O6WB, O7WC, O8W); and 0.3 (O5W, O6WA, O7WA, O7WB).
EtOH: 0.4
Fully occupied chloride: 1.0 (Cl1)

[d] *Cg*1 = D-ring (O13X–C12X–C16X–O15X–C14X)
*Cg*2 = C-ring (C9X–C10X–C11X–C12X–C16X–C17X)
*Cg*3 = B-ring (C18X–C19X–C20X–C21X–C22X–C23X)

(b) π···π interactions in β-CD·PXT·HCl·0.4EtOH·7H_2O [Å, °].

Cg(*I*)	*Cg*(*J*)	*Cg*–*Cg*	Alpha	*CgI*_Perp	*CgJ*_Perp	Type
*Cg*1	*Cg*3(*x* – 1, *y*, *z*)	5.595(6)	69.8(7)	1.938(6)	3.790(3)	Edge-to-face
*Cg*3	*Cg*2(*x*, *y*, *z*)	5.457(3)	65.7(3)	0.343(3)	4.799(3)	Edge-to-face

Note:
- *Cg*(*I*) = Plane number *I*; for *Cg*1, *Cg*2, and *Cg*3, see above.
- Alpha = Interplanar angle between planes *I* and *J* (°)
- *Cg*–*Cg* = Distance between ring centroids (Å)
- *CgI*_Perp = Perpendicular distance of *Cg*(*I*) on ring *J* (Å)
- *CgJ*_Perp = Perpendicular distance of *Cg*(*J*) on ring *I* (Å)

II. Computational data

Table S4. Hydrogen bond parameters in 1:1 and 2:1 β-CD–PXT inclusion complexes from DFT full-geometry optimization [Å, º]. [a]

D–H···A	D–H	H···A	D···A	∠(DHA)	D–H···A	D–H	H···A	D···A	∠(DHA)
1:1 [b]			β–CD	conformation					
β-*CD*–β-*CD*			Distorted	round	β-*CD*–*PXT*				
O21–H···O37	0.98	2.05	2.98	157.8	O35–H···O13X	0.98	1.97	2.92	162.3
O31–H···O22	0.98	1.99	2.95	166.9	O25–H···*Cg*2(C) [c]	0.98	3.74	4.34	121.8
O32–H···O23	0.98	1.96	2.92	167.4	C31–H···*Cg*3(B)	1.10	3.66	4.74	165.3
O62–H···O53	0.97	2.14	3.02	149.2					
O33–H···O24	0.98	1.86	2.82	165.7					
O25–H···O34	0.98	1.88	2.86	175.0					
O26–H···O35	0.99	1.87	2.84	168.3					
O27–H···O36	0.98	1.93	2.89	164.5					
2:1 [b]									
β-*CD*–β-*CD*			Distorted	round	β-*CD*–*PXT*				
O21_1–H···O37_1	0.99	1.82	2.81	171.1	O61_2–H···N1P	0.99	1.91	2.90	172.8
O22_1–H···O31_1	0.98	2.10	2.99	150.8	C36_1–H···*Cg*2(C) [c]	1.10	3.57	4.65	168.3
O32_1–H···O37_2	0.99	1.78	2.75	166.5	C36_2–H···*Cg*3(B)	1.10	3.50	4.55	161.1
O23_1–H···O32_1	0.98	1.97	2.90	155.9					
O33_1–H···O36_2	0.99	1.89	2.88	174.6					
O24_1–H···O33_1	0.99	1.79	2.78	177.1					
O34_1–H···O25_1	0.99	1.82	2.79	166.8					
O25_1–H···O34_2	0.98	1.91	2.76	143.5					
O35_1–H···O24_2	0.99	1.80	2.72	154.2					
O65_1–H···O56_1	0.97	2.07	2.96	149.8					
O26_1–H···O35_1	0.99	1.84	2.82	169.7					
O36_1–H···O27_1	0.99	1.87	2.85	175.5					
O66_1–H···O67_1	0.98	2.14	3.05	153.5					
O27_1–H···O32_2	0.99	1.85	2.73	148.2					
O37_1–H···O22_2	0.99	1.79	2.73	157.9					
O31_2–H···O31_1	0.98	1.95	2.92	166.0					
O22_2–H···O31_2	0.99	1.87	2.84	166.2					
O32_2–H···O23_2	0.99	1.79	2.77	169.3					
O23_2–H···O36_1	0.98	1.85	2.72	145.1					
O33_2–H···O26_1	0.99	1.77	2.71	156.0					
O24_2–H···O33_2	0.99	1.82	2.80	170.1					
O34_2–H···O25_2	0.99	1.78	2.77	172.3					
O25_2–H···O34_1	0.98	1.91	2.78	146.7					
O35_2–H···O24_1	0.99	1.71	2.68	167.6					
O26_2–H···O35_2	0.98	1.92	2.89	169.9					
O36_2–H···O27_2	0.98	2.01	2.97	165.0					
O37_2–H···O21_2	0.98	1.88	2.85	169.5					
O67_2–H···O51_2	0.97	2.17	2.97	138.7					

[a] DFT energy minimization in vacuum at the B3LYP/6–31+G*/4–31G level, see also Table S5.
[b] X-ray structures of the 1:1 β-CD–PXT (form II) and 2:1 β-CD–PXT (form I; [3]) with PXT in neutral form were used as starting models.
[c] PXT base in the 1:1 and 2:1 β-CD–PXT inclusion complexes, which are respectively labeled X and P
*Cg*1 = D-ring (O13X–C12X–C16X–O15X–C14X)
*Cg*2 = C-ring (C9X–C10X–C11X–C12X–C16X–C17X)
*Cg*3 = B-ring (C18X–C19X–C20X–C21X–C22X–C23X)
*Cg*1 = D-ring (O13P–C12P–C16P–O15P–C14P)
*Cg*2 = C-ring (C9P–C10P–C11P–C12P–C16P–C17P)
*Cg*3 = B-ring (C18P–C19P–C20P–C21P–C22P–C23P)

Table S5. Stabilization and interaction energies of 1:1 and 2:1 β-CD–PXT inclusion complexes from DFT full-geometry optimization. [a]

	1:1 [b]	2:1 [b]
E_{cpx} [c]	−5388.30855	−9661.36284
$E_{β\text{-}CD\_opt}$ [d] {$E_{2β\text{-}CD\_opt}$}	−4272.96405	{−8546.03560}
E_{D_opt}	−1115.32034	−1115.31793
$E_{β\text{-}CD\_sp}$ {$E_{2β\text{-}CD\_sp}$}	−4272.96123	{−8546.02372}
E_{D_sp}	−1115.31830	−1115.31277
$ΔE_{stb}$ [Hartree] [e]	−0.02401	−0.00931
$ΔE_{stb}$ [kcal mol^{-1}]	−15.16	−5.84
$ΔE_{int}$ [Hartree] [f]	−0.02902	−0.02635
$ΔE_{int}$ [kcal mol^{-1}]	−18.21	−16.54
Host-guest interactions	O–H···O, C/O–H···π	O–H···N, C–H···π

[a] DFT/B3LYP calculations in the gas phase with mixed basis sets 4–31G for C atoms and 6–31+G* for H, N, O, F atoms were carried out using program GAUSSIAN09 [4].
[b] X-ray structures of the 1:1 β-CD–PXT (form II) and 2:1 β-CD–PXT (form I; [3]) with PXT in neutral form were used as starting models, see also Tables S4 and S6.
[c] Original unit of E is Hartree [1 H = 627.5 kcal mol^{-1}].
[d] $E_{β\text{-}CD\_opt}$ in vacuum of the uncomplexed β-CD·12H_2O [5] is −4272.96662 H.
[e,f] Stabilization energy, $ΔE_{stb} = E_{cpx} − (E_{β\text{-}CD\_opt} + E_{D\_opt})$ and interaction energy, $ΔE_{int} = E_{cpx} − (E_{β\text{-}CD\_sp} + E_{D\_sp})$, where E_{cpx}, $E_{β\text{-}CD\_opt}$ and E_{D_opt} are the molecular energies from full optimization of complex, host β-CD and drug PXT in the free base form, respectively; $E_{β\text{-}CD\_sp}$ and E_{D_sp} are the corresponding single-point energies in the complexed states.

Table S6. Dispersion- and BSSE-corrected interaction energies of 1:1 and 2:1 β-CD–PXT inclusion complexes from DFT/B97D calculations. [a]

	1:1	2:1
B97D		
E_{cpx}	−5385.02492	−9655.57586
$E_{β\text{-}CD\_sp}$ {$E_{2β\text{-}CD\_opt}$}	−4270.36709	{−8540.87255}
E_{D_sp}	−1114.58685	−1114.58275
$ΔE_{int}$ [Hartree]	−0.07098	−0.12056
$ΔE_{int}$ [kcal mol^{-1}]	−44.54	−75.65
$ΔΔE_{int}$ [kcal mol^{-1}] [d]	31.12	0.00
B97D+BSSE		
E_{cpx_BSSE}	−5385.00925	−9655.54649
$E_{β\text{-}CD(cpx)\_sp}$ {$E_{2β\text{-}CD(cpx)\_sp}$}	−4270.37175	{−8540.88338}
$E_{D(cpx)\_sp}$	−1114.59786	−1114.60128
$ΔE_{BSSE}$ [Hartree] [b]	0.01567	0.02937
$ΔE_{BSSE}$ [kcal mol^{-1}] (% contribution to $ΔE_{int}$)	9.83 (22%)	18.43 (24%)
$ΔE_{int\_BSSE}$ [Hartree] [c]	−0.05531	−0.09120
$ΔE_{int\_BSSE}$ [kcal mol^{-1}]	−34.71	−57.23
$ΔΔE_{int\_BSSE}$ [kcal mol^{-1}] [d]	22.52	0.00

[a] DFT/B97D calculations in the gas phase with mixed basis sets 4−31G for C atoms and 6−31+G* for H, N, O, F atoms were carried out using program GAUSSIAN09 [4]. DFT/B3LYP-optimized structures were used for single-point energy calculations, see also Table S7.
[b] The basis set superposition error (BSSE) energy by counterpoise correction [6],
$ΔE_{BSSE} = E_{cpx\_BSSE} − E_{cpx} = [E_{β\text{-}CD\_sp} − E_{β\text{-}CD(cpx)\_sp}] + [E_{D\_sp} − E_{D(cpx)\_sp}]$
where E_{cpx_BSSE} and E_{cpx} are corrected and uncorrected complex energies, respectively.
[c] BSSE-corrected interaction energy, $ΔE_{int\_BSSE} = E_{cpx} − (E_{β\text{-}CD(cpx)\_sp} + E_{D(cpx)\_sp}) = ΔE_{int} + ΔE_{BSSE}$ where $E_{β\text{-}CD(cpx)\_sp}$ and $E_{D(cpx)\_sp}$ are energies of two components in the complex.
[d] Relative interaction energies ($ΔΔE_{int}$ and $ΔΔE_{int\_BSSE}$) compared to the 2:1 β-CD–PXT complex [3].

III. References

1. Cremer, D.T.; Pople, J.A. General definition of ring puckering coordinates. *J. Am. Chem. Soc.* **1975**, *97*, 1354–1358. https://doi.org/10.1021/ja00839a011.
2. French, A.D.; Johnson, G.P. Linkage and pyranosyl ring twisting in cyclodextrins. *Carbohydr. Res*. **2007**, *342*, 1223–1237. https://doi.org/10.1016/j.carres.2007.02.033.
3. Caira, M.R.; De Vries, E.; Nassimbeni, L.R.; Jacewicz, V.W. Inclusion of the antidepressant paroxetine in β-cyclodextrin. *J. Incl. Phenom. Macrocycl. Chem*. **2003**, *46*, 37–42. https://doi.org/10.1023/A:1025622809025.
4. Frisch, M.J.E.A.; Trucks, G.W.; Schlegel, H.B.; Scuseria, G.E.; Robb, M.A.; Cheeseman, J.R.; ... Nakatsuji, H. GAUSSIAN09, Revision A.01. Gaussian. Inc.; Wallingford, CT, 2009.
5. Lindner, K.; Saenger, W. Crystal and molecular structure of cyclohepta-amylose dodecahydrate. *Carbohydr. Res*. **1982**, *99*, 103–115. https://doi.org/10.1016/S0008-6215(00)81901-1.
6. Boys, S.F.; Bernardi, F.J.M.P. The calculation of small molecular interactions by the differences of separate total energies. Some procedures with reduced errors. *Mol. Phys*. **1970**, *19*, 553–566. https://doi.org/10.1080/00268977000101561.
